# Size‐dependent movement explains why bigger is better in fragmented landscapes

**DOI:** 10.1002/ece3.4524

**Published:** 2018-10-23

**Authors:** Jasmijn Hillaert, Thomas Hovestadt, Martijn L. Vandegehuchte, Dries Bonte

**Affiliations:** ^1^ Department of Biology Terrestrial Ecology Unit Ghent University Ghent Belgium; ^2^ Department of Animal Ecology and Tropical Biology Biocenter University of Wuerzburg Wuerzburg Germany

**Keywords:** allometric scaling, body size distributions, eco‐evolutionary dynamics, habitat fragmentation, isolation, metabolic theory, optimal size, size‐dependent movement

## Abstract

Body size is a fundamental trait known to allometrically scale with metabolic rate and therefore a key determinant of individual development, life history, and consequently fitness. In spatially structured environments, movement is an equally important driver of fitness. Because movement is tightly coupled with body size, we expect habitat fragmentation to induce a strong selection pressure on size variation across and within species. Changes in body size distributions are then, in turn, expected to alter food web dynamics. However, no consensus has been reached on how spatial isolation and resource growth affect consumer body size distributions. Our aim was to investigate how these two factors shape the body size distribution of consumers under scenarios of size‐dependent and size‐independent consumer movement by applying a mechanistic, individual‐based resource–consumer model. We also assessed the consequences of altered body size distributions for important ecosystem traits such as resource abundance and consumer stability. Finally, we determined those factors that explain most variation in size distributions. We demonstrate that decreasing connectivity and resource growth select for communities (or populations) consisting of larger species (or individuals) due to strong selection for the ability to move over longer distances if the movement is size‐dependent. When including size‐dependent movement, intermediate levels of connectivity result in increases in local size diversity. Due to this elevated functional diversity, resource uptake is maximized at the metapopulation or metacommunity level. At these intermediate levels of connectivity, size‐dependent movement explains most of the observed variation in size distributions. Interestingly, local and spatial stability of consumer biomass is lowest when isolation and resource growth are high. Finally, we highlight that size‐dependent movement is of vital importance for the survival of populations or communities within highly fragmented landscapes. Our results demonstrate that considering size‐dependent movement is essential to understand how habitat fragmentation and resource growth shape body size distributions—and the resulting metapopulation or metacommunity dynamics—of consumers.

## 1 INTRODUCTION

Body size sets limits to the functioning of individuals, thereby affecting inter‐ and intraspecific interactions and regulating overall food web structure (Bartholomew, 1982; Brose, Williams, & Martinez, 2006). Body size is also central to metabolic theory (Brody, 1945; Brody, Procter, & Ashworth, 1934; Brown, Gillooly, Allen, Savage, & West, 2004; Kleiber, 1932). Starting from the simple allometric rule linking body size with metabolic rate, important inferences can be made at the level of individuals, populations, communities, and ecosystems (Brown et al., 2004). For instance, the ingestion rate and speed of movement of an individual are correlated with its body size (Peters, 1983). Also, shifts in body size structure of communities have been shown to affect ecosystem functioning (Fritschie & Olden, 2016; Yvon‐Durocher & Allen, 2012). Hence, body size can be considered a super trait (Brose et al., 2017; Fritschie & Olden, 2016) relating to both ecological effects and responses and therefore constraining ecological and evolutionary dynamics (Applebaum, Pan, Hedgecock, & Manahan, 2014; Llandres et al., 2015).

Body size is directly related to individual biomass. With increasing resource productivity, more total consumer metabolic biomass can be supported (Atkins, Griffin, Angelini, O'Connor, & Silliman, 2015). However, for a given amount of resources, higher abundance implies lower per capita energy use (i.e., the energetic equivalence rule). Therefore, increased productivity can either result in more or larger individuals (Damuth, 1981; Ehnes et al., 2014; White, Ernest, Kerkhoff, & Enquist, 2007). Furthermore, resources are usually not homogeneously distributed across space, but spatially structured (Krummel, Gardner, Sugihara, O'Neill, & Coleman, 1987). This implies that organisms need to move both within (foraging resulting in spatially coupled patches) and across generations (dispersal resulting in metapopulation dynamics) to make optimal use of resources, depending on the spatiotemporal dynamics of both the resource and the consumer population (Amarasekare, 2008). Because body size determines to a large extent the movement capacity of active dispersers (Stevens et al., 2014), we expect it to have a large impact on population and community dynamics in spatially structured environments and to be under strong selection.

The cost of movement is highly dependent on resource availability and habitat connectivity, and is one of the costs that change with body size (Bonte et al., 2012; Peters, 1983). Large‐sized individuals may, for instance, incur higher costs due to their larger home ranges but more directly, we expect larger body sizes to be associated with reduced time costs because of higher achieved speed and increased perceptual range (Buddenbrock, 1934; Mech & Zollner, 2002; Pawar, Dell, & Savage, 2012; Peters, 1983). This view is at the basis of the textural discontinuity hypothesis, which states that the modes of a size abundance distribution mirror those scales at which resources within the landscape are most abundant, relative to the size and movement capacity of the consumer species (Borthagaray, Arim, & Marquet, 2012; Holling, 1992; Nash et al., 2014). Despite the attention of theoretical studies to the origin of size distributions (e.g., Loeuille & Loreau, 2005; Ritterskamp, Bearup, & Blasius, 2016), only few have covered the dependence of size distributions on habitat configuration (but see, Milne, Turner, Wiens, & Johnson, 1992; Etienne & Olff, 2004; Borthagaray et al., 2012; Buchmann, Schurr, Nathan, & Jeltsch, 2012, 2013). Overall, theory based only on spatial scaling of size versus resource distribution predicts that resource availability and distribution strongly affect body size distributions of species within communities (Allen et al., 2006; Borthagaray et al., 2012; Holling, 1992; Nash et al., 2014; Ritchie & Olff, 1999).

Body size is not only central to mobility and metabolic rate, but also to development (West, Brown, & Enquist, 2001). Small individuals and species have the advantage of low energy requirements and short developmental times (Peters, 1983). Large individuals and species, on the other hand, are capable of crossing unsuitable matrix to reach new patches and have higher tolerances to starvation (Davies, Margules, & Lawrence, 2000; Peters, 1983; Tscharntke & Brandl, 2004). This could explain why, although many empirical studies have investigated the effect of habitat fragmentation on body size distributions, a conclusive pattern remains elusive (e.g., Davies et al., 2000; Hamback et al., 2007; Jauker, Speckmann, & Wolters, 2016; Renauld, Hutchinson, Loeb, Poveda, & Connelly, 2016; Warzecha, Diekötter, Wolters, & Jauker, 2016). It thus remains difficult to predict how the spatial distribution of resources affects body size distributions.

As body size is central to both movement and resource consumption, its distribution in space and time is expected to have a strong impact on ecosystem stability, primary productivity, and biodiversity (Massol et al., 2017). Individuals in metapopulations or metacommunities function as mobile linkers that organize themselves in space to maximize their fitness according to their size (Jeltsch et al., 2013). Further, stabilizing mechanisms may allow for species coexistence, increasing diversity, which has been shown to be positively affected by habitat fragmentation (Arnillas, Tovar, Cadotte, & Buytaert, 2017; Fahrig, 2017; Jeltsch et al., 2013). Variation in the body size of consumers and the connectivity of their habitat are also expected to alter resource abundance. Resource abundance will in turn alter consumer biomass, regulating ecosystem stability, which is crucial for ecosystem functioning and sustainability and varies across scales (Wang & Loreau, 2014).

Due to the ubiquitous increase in habitat loss and fragmentation, we urgently need to better understand communities’ and species’ responses to isolation. Fine‐grained fragmentation has been shown to have a large effect on reproduction and survival (Cattarino, Mcalpine, & Rhodes, 2016). Still, most research is performed at the large spatial and temporal scales of metacommunity dynamics (e.g., Davies et al., 2000; exceptions: Buchmann et al., 2013; Braschler & Baur, 2016). Because current theory fails to formally link selection on body size to metabolic and metapopulation theory, we applied an individual‐based, spatially explicit model to study the effect of fine‐grained resource isolation on the selection on body size distributions of a consumer population or community. As habitat isolation might affect a resource's growth speed by changing abiotic factors, we also test how resource growth speed interacts with habitat isolation in affecting the selection on body size. We based our model on established allometric rules linking body size to movement speed, movement costs, basal metabolic rate, ingestion rate, growth rate, and reproduction. The development of such a complex, mechanistic model is a necessity when studying body size as these allometric rules imply the possibility of crucial trade‐offs, which should not be overlooked. As these rules are universal and apply to a wide range of taxa (ectotherms and endotherms), our results are applicable to any organism that moves actively and grows deterministically (Brown et al., 2004; Peters, 1983). Importantly, as we focus on the effect of fragmentation at the scale of foraging (fine‐grained fragmentation), any extension is possible as long as the relative scale of fine‐grained fragmentation is updated to the scale of foraging of the species or community of interest. We further aim to uncover the importance of size‐dependent movement for selection on consumer body size and ecological dynamics of resource and consumer populations. Moreover, the impact on crucial ecosystem traits such as ecosystem stability at various scales and resource abundance is estimated. Our individual‐based approach enables us to interpret our results either as within‐species adaptive dynamics of individual body size or across‐species metacommunity changes in the distribution of species of different size. Overall, we expect an increasing importance of size‐dependent movement in environments with increasing isolation of the resources and thus a (community‐wide) shift toward larger body sizes as habitat fragmentation increases.

## 2 Methodology

We modified the consumer–resource model presented in Hillaert, Vandegehuchte, Hovestadt, and Bonte (2018) to understand how the spatial distribution of a resource affects the size distribution of its consumer(s). The spatial distribution of the resource and its abundance differed between simulations with regard to the distance between its suitable patches (nearest neighbor distance [*NND*)] and its growth rate. This resource may be consumed by (a community of) consumers. All traits of the consumers are related to their mass by allometric rules, as derived from the literature (e.g., Peters, 1983). An individual's body mass is used to represent its size (Peters, 1983). Also, we assessed the importance of size‐dependent movement for shaping the evolved consumer size distribution and its impact on metapopulation functioning. This was done by creating two models: a coupled and a decoupled model. In the coupled model, speed of movement and perceptual range both increase with body size, whereas in the decoupled model, body size, perceptual range, and speed of movement are unlinked.

The model is a spatially explicit, discrete‐time model with overlapping generations. One time step corresponds to one day within the lifetime of the consumer. We here took an arthropod‐centered angle and parameterized allometric rules for a haploid, parthenogenetic arthropod species feeding on plants (the resource) with a semelparous life cycle. See Supporting Information Appendix S4 for a detailed description of our model following the overview, design concepts, and details (ODD) protocol (Grimm et al., 2006, 2010). All parameters of the model and their default values are summarized in Supporting Information Table S4.1 in Appendix S4.

### 2.1 The landscape

The landscape is cell‐based with each cell having a side length (*SL*) of 0.25 m. Within the landscape, a distinction is made between suitable and unsuitable habitat. Resources only grow within suitable patches with one patch having the size of a single cell. All landscapes have a constant number of suitable patches (i.e., 2,500) but varying *NND* (Fahrig, 2003). The effect of isolation is tested by assigning a constant *NND* from 0 to 10 to all cells (see Supporting Information Appendix S3 for an example). Consequently, the dimensions of the landscape increase with *NND* according to (50 + NND*50) × (50 + NND*50) cells. The boundaries of the landscape are wrapped.

### 2.2 The resource

As it is advisable not to focus on individual species but also cover their interactions with other species (Berg et al., 2010), we included the dependence of the consumer on its resource by varying the resource's growth speed. Resources at the cell level are not individually modeled but by a local logistic growth model. Local resource biomass is represented as the total energetic content of resource tissue within that cell (*R*_*x,y*_ in Joules). This resource grows logistically in time depending on the resource's carrying capacity (*K*) and intrinsic growth rate (*r*). *K* was set to 2,000 *J* (assumption of space limitation), whereas *r* differed between simulations (0.1, 0.5, or 0.9 per day; assumption for the productivity of the system). In any cell, a fixed amount of resource tissue (*E*_nc_, in Joules, fixed at *1* *J*) is nonconsumable by the consumer species, representing belowground plant parts. As such, *E*_nc_ is the minimum amount of resource tissue present within a suitable cell, even following local depletion by the consumer species.

### 2.3 The consumer

**Figure 1:**
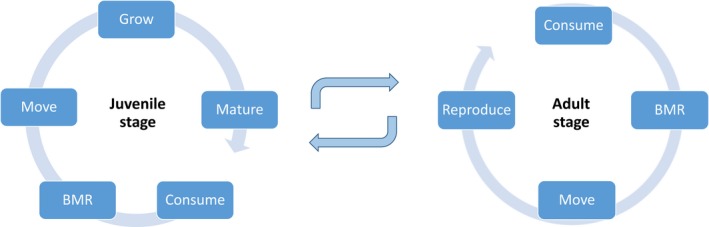
A comparison of daily events for the juvenile and adult stage of the consumer (Hillaert et al., 2018). BMR stands for the basal metabolic rate costs

All consumers are individually modeled within the landscape. The consumer has two life stages: a juvenile and adult life stage. Within a day, both stages have the chance to execute different events (see Figure 1).

First, an individual nourishes its energy reserve by consumption. Second, the energy reserve is depleted by the cost of daily maintenance (i.e., basal metabolic rate). Third, an individual has the opportunity to move. Fourth, juveniles may grow, eventually resulting in maturation, if they approximate their adult mass (*W*_max_). Energy for reproduction is collected during several days as only one clutch is produced during the lifetime of an individual. The energetic threshold for reproduction increases with body size.

Moreover, the energy of an individual's energy reserve (*E*_*r*_) is invested in the following order: (a) basal metabolic rate, (b) movement, and (c) growth or reproduction. As such, an individual's priority is investing energy in the basal metabolic rate cost. Secondly, it will try to guarantee access to resources the following day by moving. Thirdly, it will invest remaining energy in growth or reproduction if some energy is left. As the consumer species is semelparous, adults die after reproduction.

Energy from consumed resources that were not expended during a day remains in the energy reserve. Body size is linked to many features of an individual. In this model, larger individuals move faster, have longer developmental times, larger clutch sizes, higher basal metabolic rates and higher ingestion rates. These traits also change during the developmental phase of an individual, corresponding to its body mass.

Individual body size at maturity (*W*_max_, in kg) is coded by a single gene. Adult size is heritable and may mutate with a probability of 0.001 during reproduction. This mutation rate is commonly applied within theoretical models (Henry, Coulon, & Travis, 2015; Travis, Mustin, Benton, & Dytham, 2009). A new mutation is drawn from the uniform distribution [*W*_max_ –* *(*W*_max_/2), *W*_max_ + (*W*_max_/2)] with *W*_max_ referring to the adult size of the parent. New mutations may not exceed the predefined boundaries [0.01 g, 3 g] that represent absolute physiological limits. As such, our minimum adult size corresponds to the size of a small grasshopper such as *Tetrix undulata* (0.01 g) and the maximum size (3 g) to that of some longhorn beetles (Cerambycidae), darkling beetles (Tenebrionidae), scarab beetles (Scarabaeidae), or grasshoppers (Acrididae). New variants of this trait may also originate by immigration (see below). Mutation enables fine‐tuning of the optimal body size, whereas immigration facilitates fitness peak shifts.

### 2.4 Initialization

Per parameter combination, 10 simulations were run. At the start of each simulation, 1,000 adult individuals were placed into the landscape. The adult mass of each individual (*W*_max_) was defined as 10 raised to the power of a value drawn from the uniform interval [−5, −2.522878745]. In other words, we sample a value between 0.00001 kg (minimum adult mass) and 0.003 kg (maximum adult mass). As such, individuals with masses of different orders of magnitude have an equal chance of being initialized in the landscape. Moreover, initialized distributions are skewed toward small individuals. Also, each initialized individual carried enough energy within its energy reserve to survive the first day. This amount of energy is calculated based on an individual's mass and accounts for the cost of basal metabolic rate and movement during one day. Initial resource availability per cell corresponded to the maximum carrying capacity.

### 2.5 Immigration

The frequency with which immigrants arrive in the landscape is described by *q*. This variable is fixed at one per 10 days. The process of determining an immigrant's adults mass is similar as during initialization. An immigrant is always introduced within a suitable cell, and its energy reserve contains just enough energy to survive the first day. This amount of energy is calculated based on an individual's mass and accounts for the cost of basal metabolic rate and movement during one day.

### 2.6 Consumer events

How body size affects all consumer events is explained in detail in the ODD description of our model (see Supporting Information Appendix S4). Here, we give a short overview of the events and their most important equations.

#### 2.6.1 Consumption

The amount of energy ingested per day for an individual (*i*_max_ in Joules) is determined as

(1) $$i_{\max}=2\cdot W^{0.80}\cdot t_{f},$$

with *W* being body mass (in kg) (Peters, 1983). *t*_*f*_ refers to the time devoted per day to consumption (in seconds) and is fixed at 15 hr. Competition for resources is scramble.

#### 2.6.2 Basal metabolic rate

The standard metabolic rate of poikilotherms (*M*, in watts) is described by

(2) $$M=0.14W^{0.751}$$

(Hemmingsen, 1960 cited in Peters, 1983).

#### 2.6.3 The movement phase

##### 2.6.3.1 Probability of moving (*p*)

Whether an individual moves depends on the ratio of the amount of energy present within a cell (*R*_*x,y*_) and the maximum amount of energy that can be consumed by all consumers present within that cell. This latter factor is determined by calculating the sum of all individuals’ daily ingestion rates within that cell ($\Sigma i_{\max_{x,y}}$).

By assuming a symmetric competition, the probability of moving (*p*) is equal for all individuals present within the same cell and is calculated by (based on Poethke and Hovestadt (2002)):

(3) $$\begin{array}{cc} p=1-\frac{R_{x,y}}{\Sigma i_{\max_{x,y}}} & \text{if}\;\frac{R_{x,y}}{\Sigma i_{\max_{x,y}}}<1\\ p=0 & \text{if}\;\frac{R_{x,y}}{\Sigma i_{\max_{x,y}}}\geq 1 \end{array}.$$

##### 2.6.3.2 Defining searching area

As one time step in our model corresponds to one day, we do not model the movement behavior of an individual explicitly but instead, estimate the total area an individual can search for resources during a day. This area is called an individual's searching area and is calculated once per time step, for each moving individual. As all cells at a particular distance from the origin are equally intensively searched, the searching area is circular with a radius (*rad*) and a center corresponding to the current location of an individual (Delgado, Barton, Bonte, & Travis, 2014). An individual's searching area increases with an individual's optimal speed (*v*_opt_), movement time (*t*_m_), and perceptual range (*d*_per_). Both optimal speed and perceptual range depend on body mass, resulting in larger searching areas for larger individuals. The cost of movement includes the energy invested by an individual in prospecting its total searching area. Therefore, it is dependent on the size of the total searching area instead of the shortest distance between the cell of origin and cell of destination.

An individual's average speed of movement (*v*_opt_, in meters per second) is calculated by means of the following allometric equation, derived for walking insects (Buddenbrock, 1934 cited in Peters, 1983):

(4) $$v_{\mathrm{opt}}=0.3\cdot W^{0.29}.$$

Here, *W* refers to the mass of an individual in kg, ignoring the mass of stored resources. The time an individual invests in movement per day (*t*_m_, in seconds) is maximally 1 hr. In case too little internally stored energy (*E*_r_) is present to support the movement for 1 hr, *t*_m_ is calculated by:

(5) $$t_{m}=\frac{E_{r}}{c_{m}}.$$

*c*_m_ refers to the energetic cost of movement (in Joules per second) and is calculated by the following formula, which is based on running poikilotherms (Buddenbrock, 1934 cited in Peters, 1983):

(6) $$c_{m}=\left(0.17W^{0.75}+3.4W\right).$$

The cost of moving during the time *t*_m_ (*t*_m_ · *c*_m_) is subtracted from an individual's energy reserve. Based on *t*_m_ and *v*_opt_, the total distance an individual covers at day *t* (*d*_max_) is determined:

(7) $$d_{\max}=v_{\mathrm{opt}}\cdot t_{m}.$$

Next, the perceptual range of an individual is determined by means of the following relationship:

(8) $$d_{\mathrm{per}}=301W+0.097.$$

For simplicity, this relationship is linear and based on the assumption that the smallest individual (0.01 g) has a perceptual range of 0.10 m and the largest individual (3 g) a perceptual range of 1 m. The effect of this relationship has been tested (see sensitivity analysis, Supporting Information Appendix S5). Moreover, the positive relationship between body size and perceptual range or reaction distance has been illustrated over a wide range of taxa, including arthropods (supplementary information of Pawar et al., 2012).

The searching area of an individual is circular, and its radius (*rad*, in m) is calculated by taking into account the total distance the individual has covered during the day and the individual's perceptual range (see Supporting Information Appendix S2 for an explanation of the formula calculating *rad*).

##### 2.6.3.3 Habitat choice

As habitat choice is informed, an individual moves to the cell with the highest amount of resources within its searching area.

#### 2.6.4 Growth

The applied growth model is the one described by West et al. (2001) for deterministic growth.

#### 2.6.5 Maturation

Juveniles reaching 99% of their adult mass (*W*_max_) mature.

#### 2.6.6 Reproduction

During reproduction, the relationship between total clutch size (CS, in kg) and mass (*W*, in kg) is determined by the following equation which is based on aquatic poikilotherms (Blueweiss et al., 1978):

(9) $$\text{CS}=0.158W^{0.92}.$$

For simplicity, the number of eggs per clutch (*N*) is fixed at 15.

### 2.7 Coupled versus decoupled model

To determine the importance of size‐dependent movement, two different models were created: a coupled and a decoupled model. In the coupled model, speed of movement (*v*_opt_) and perceptual range both increase with body size. The decoupled model represents a null model in which body size, speed of movement, and perceptual range were unlinked. Body size and speed of movement were unlinked by resampling an individual's speed of movement each day from the uniform range [0.0106, 0.0557]. Here, 0.0106 corresponds to the optimal speed of the smallest adult individual (0.01 g) and 0.0557 to the optimal speed of the largest adult individual (3 g). Also, the perceptual range of an individual is no longer increasing with body size, but instead sampled daily from the uniform distribution [0.1 m, 1 m]. 0.1 m corresponds to the perceptual range of the smallest adult individual (0.01 g) and 1 m to the perceptual range of the largest adult individual (3 g). We chose to sample from a uniform distribution rather than from an evolved scenario in the decoupled model to avoid any skewness and bias in the randomization. As the cost of movement is based on the total movement time and not total distance, it is unaffected by the decoupling.

### 2.8 Data analysis

Within the coupled model, the simulations with *NND* 10 and growth speed of 0.1 and 0.5 went extinct without immigration from outside the landscape; therefore, we omitted these simulations during the analysis.

During each simulation, we traced changes in the mean amount of resources per cell, total number of adults and juveniles, average adult weight (*W*_max_), and the coefficient of variation, skewness, and kurtosis of the consumer's adult weight (*W*_max_) distribution. Every 500 time steps, the value of *W*_max_ of maximum 50,000 randomly sampled individuals was collected.

#### 2.8.1 Occupancy

Occupancy (*O*) is defined as the ratio of occupied patches to the total number of suitable patches within the landscape. The level of occupancy is determined every 10 days during the last 100 days of a simulation. In the end, the average of these values is calculated per simulation.

#### 2.8.2 Variability

In order to infer the stability of the community at several scales, we calculated the *α*,* β*_2_, and *γ* variability per simulation. *α* variability is a measure of the local temporal variability and is calculated as:

(10) $$\alpha_{\mathrm{CV}}={\left(\frac{\sum_{m}\sqrt{w_{m}}}{\sum_{m}\mu_{m}}\right)}^{2},$$

with *w*_*m*_ referring to the temporal variance and *μ*_*m*_ to the temporal mean of community consumer biomass in cell *m* (Wang & Loreau, 2014). The temporal variability at the metacommunity scale or *γ* variability was calculated as:

(11) $$\gamma_{\mathrm{CV}}=\frac{\sum_{m,n}w_{\mathrm{mn}}}{{\left(\sum_{m}\mu_{m}\right)}^{2}},$$

with *w*_*mn*_ referring to the temporal covariance of community biomass between cells *m* and *n* (Wang & Loreau, 2014). Finally, *β*_2_ variability or asynchrony‐related spatial variability was determined as:

(12) $$\beta_{2}=\alpha_{\mathrm{CV}}-\gamma_{\mathrm{CV}}.$$

In order to calculate these variables, we recorded the total consumer biomass of 100 randomly selected suitable patches every 10 days during the final 100 days of a simulation.

#### 2.8.3 Reproductive success and movement

Throughout the final 600 days of a simulation, 1,000 eggs were randomly selected to be followed during their lifetime. Their movements and reproductive success were recorded.

#### 2.8.4 Variation partitioning

By means of multivariate variation partitioning, we disentangled the amount of variation in adult size that can be explained by the coupling of body size and movement, resource growth rate, and level of isolation. Analysis was performed in R by applying the function varpart within the package vegan which is based on calculating the adjusted *R* ^*2*^ in redundancy analysis ordination (Oksanen et al., 2018). Variation partitioning analyses were performed on the average, coefficient of variation, level of skewness, and level of kurtosis of the distribution of *W*_max_, collected per simulation. We also executed a similar analysis for (a) occupancy, (b) parameters summarizing resource and consumer dynamics (resource abundance, resource variance, and consumer abundance), and (c) the metapopulation functioning statistics *α*,* β*_2_, and *γ* variability. We executed a global variation partitioning including all distances except for *NND* 10 as some of these simulations were not stable and only survived as sinks. We furthermore executed a variation partitioning for each value of *NND* independently. As such, the effect of isolation on the amount of variation explained by the coupling of body size and movement could be estimated. In order to guarantee that each parameter contributed equally, all data were *z*‐transformed prior to analysis.

## 3 RESULTS

### 3.1 The coupled model

**Figure 2:**
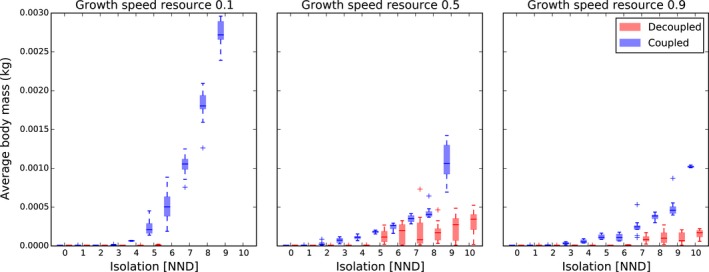
Effect of isolation and resource growth speed on the average adult body mass (*W*_max_) of a consumer. In the coupled model, movement is dependent on body size, while in the decoupled model, both are independent. *NND*: nearest neighbor distance expressed in number of cells

**Figure 3:**
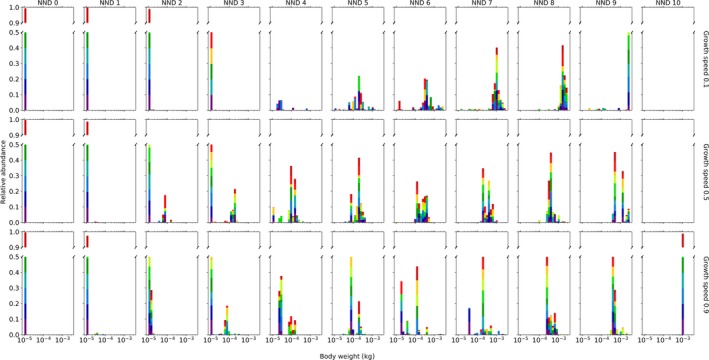
A detailed overview of the evolved adult body size (*W*_max_) distribution of a consumer feeding on a resource when movement is dependent on body size (coupled model). The body size distribution of the consumer clearly depends on the degree of isolation within the landscape (*NND*: nearest neighbor distance) and growth speed of its resource. Per scenario, 10 simulations were run. Each simulation is displayed in a different color

Consumers evolve a larger body size with increasing rates of isolation (Figures 2 and 3). The effect of isolation is additionally strengthened under conditions where resource growth speed is reduced (Figure 2). When isolation is low, adult body size distributions are right skewed with high kurtosis, whereas with increasing isolation, these distributions become more neutrally skewed with low kurtosis (Supporting Information Figures S1.1 and S1.2 in Appendix S1). Selection of increasing consumer body size with increasing patch isolation is associated with low consumer abundances (Supporting Information Figures S1.3 in Appendix S1) and rare but far movements (Supporting Information Figures S1.4 and S1.5 in Appendix S1) relative to metapopulations with highly connected patches. As expected, the level of occupancy decreases with increasing isolation (except for simulations with a growth speed of 0.9 and *NND* of 10; Supporting Information Figure S1.6 in Appendix S1).

At intermediate levels of isolation and a growth speed of 0.5 or 0.9, body size is often distributed with two peaks within one simulation (see colors in Figure 3). In most simulations, the abundances of these optimal body sizes appear to fluctuate between these two optima, coinciding with a fluctuation in the amount of resources available within the landscape (Supporting Information Figure S1.7 in Appendix S1). At these intermediate distances, the diversity in size is largest and fluctuating. The coexistence of these multiple body sizes within a community leads to a more efficient depletion of resources within the landscape and lower individual starvation rates (Supporting Information Figures S1.8 and S1.9 in Appendix S1). Population or community‐level resource depletion is lowest when nearest neighbor distance is largest (Supporting Information Figure S1.8 in Appendix S1). When growth speed is low and isolation intermediate (*NND* 3–6), extinction is common during the initial stages of a simulation due to fast depletion of the resources and few reachable patches (Supporting Information Table S1.1 in Appendix S1). However, if a population survives this stage and reaches equilibrium, individual starvation chance is lowest (Supporting Information Figure S1.9 in Appendix S1). The simulations with a low resource growth speed have the highest *α*,* β*_2_, and *γ* variability (Supporting Information Figures S1.10–12 in Appendix S1). When growth speed is high, *α* and *β*_2_ variability decrease with increasing isolation (Supporting Information Figures S1.10 and S1.11 in Appendix S1).

### 3.2 The decoupled model

**Figure 4:**
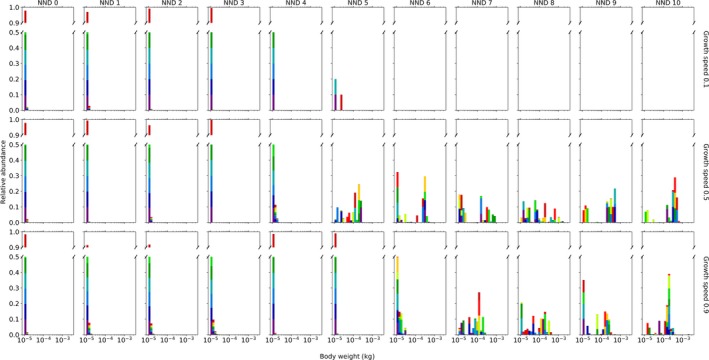
A detailed overview of the evolved optimal adult body size (*W*_max_) distribution of a consumer feeding on a resource when movement is independent of body size (the decoupled model). The effect of isolation (*NND*: nearest neighbor distance) and growth speed of the resource on the optimal body size distribution of the consumer are shown. Per scenario, 10 simulations were run. Each simulation is displayed in a different color

Importantly, all simulations with low growth speed (0.1) and high levels of isolation (more than 5 *NND*) go extinct within the decoupled model (Supporting Information Table S1.1 in Appendix S1). Generally, size‐independent movement selects for smaller average adult body sizes (Figure 2). Interestingly, when immigration of novel genotypes within the metapopulation is not allowed (*q *=* *0), adult body size converges to the minimum in almost all simulations (p5 sensitivity analysis, Supporting Information Appendix S5). In metapopulations with large nearest neighbor distances, this minimum size is not obtained when immigration is allowed (*q* = 0.1) (Figure 4). At low and intermediate levels of isolation, the smallest individuals of 0.01 g are being selected (Figures 2 and 4). Therefore, the level of kurtosis and skewness of these simulations are higher than within the coupled model (Supporting Information Figures S1.1 and S1.2 in Appendix S1). Globally, more individuals are present within the decoupled model than the coupled model (Supporting Information Figure S1.3 in Appendix S1). The number of individuals increases slightly with moderate isolation but decreases drastically at high levels of isolation (Supporting Information Figure S1.3 in Appendix S1). The average amount of resources shows an opposite pattern (Supporting Information Figure S1.8 in Appendix S1). Due to the decoupling of body size and movement, individuals move further (Supporting Information Figure S1.4 in Appendix S1). Simultaneously, the chance of moving during a day is also higher, except when isolation is low (Supporting Information Figure S1.5 in Appendix S1). As such, the total average distance moved during a lifetime is on average higher within the decoupled model (Supporting Information Figure S1.13 in Appendix S1). At high levels of isolation, the chance of dying due to starvation is remarkably lower (Supporting Information Figure S1.9 in Appendix S1), resulting in a longer lifetime (Supporting Information Figure S1.14 in Appendix S1). Due to changes in movement frequency and distance, the level of occupancy is higher within the decoupled model (Supporting Information Figure S1.6 in Appendix S1). As within the coupled model, the simulations with a growth speed of 0.1 appear to have the highest *α*,* β*_2_, and *γ* variability (Supporting Information Figures S1.10–12 in Appendix S1).

### 3.3 Partitioning variance: Importance of size‐dependent movement for selection and ecological dynamics

Most variation in adult body size distributions is explained by the level of isolation (Table 1). Resource growth speed and size‐dependent movement are less but almost equally important for the weight distribution of a consumer (Table 1). Resource growth speed explains most of the total variation in occupancy rate (*O*), consumer and resource dynamics, and metapopulation statistics *α, β*_2,_ and *γ* variability (Table 1). Moreover, the level of isolation is more important than size‐dependent movement for consumer and resource dynamics. The level of isolation only explains very little variation in *α, β*_2,_ and *γ* variability and *O* (Table 1).

**Table 1:** An overview of the amount of variation in (a) weight distribution (average, coefficient of variation, skewness, and kurtosis of *W*_max_ distribution), (b) occupancy, (c) resource and consumer dynamics (resource abundance, resource variance, and consumer abundance), and (d) metapopulation variability (*α*,* β*_2_, and *γ* variability) that can be explained by the coupling of movement and size, the level of isolation, and resource growth speed

	Coupling	Isolation	Resource growth speed
Weight distribution	0.07434	0.23488	0.09935
Occupancy	0.10450	0.03184	0.65737
Resource and consumer dynamics	0.05374	0.10723	0.28712
Metapopulation variability	0.0536	0.00069	0.36578

Of all the statistics of interest, the coupling of size and movement has the largest impact on *O* (Table 1). This coupling is able to explain about 5% of the variation in both resource and consumer dynamics and *α, β*_2,_ and *γ* variability (Table 1).

**Figure 5:**
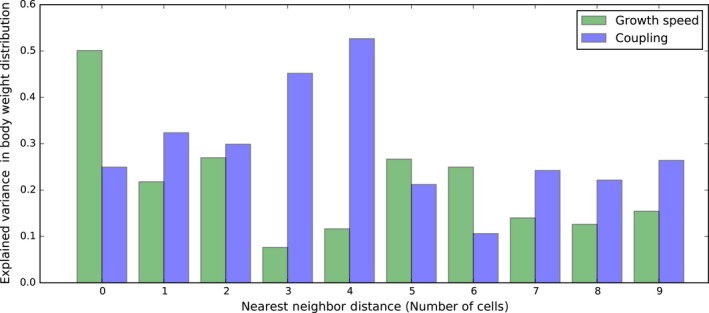
A comparison between the amount of variation in a consumer's weight distribution that is explained by growth speed and the coupling of body size and movement, for each level of isolation

The amount of variation explained by size‐dependent movement is highest at *NND* = *4*, here reaching 52.65% (Figure 5), and lower at higher and lower levels of patch connectedness (Figure 5). When isolation is strong (*NND* > 4), almost all consumer populations or communities go extinct when movement is decoupled (Supporting Information Table S1.1 in Appendix S1). Because only surviving metapopulations are integrated into the variance partitioning, less variation in body size is explained by size‐dependent movement for these levels of isolation (Figure 5). The total amount of variation in body size is additionally higher at high isolation than low or intermediate isolation within the decoupled model (Figure 2 and Supporting Information Figure S1.15 in Appendix S1).

The importance of resource growth speed in explaining variation in consumer body size is highest in the most connected landscape (50.09%) (Figure 5). For the other levels of isolation, size‐dependent movement has a higher explaining power than growth speed except for the levels of isolation with *NND* equaling 5 (26.65% vs. 21.20%) or 6 (24.94% vs. 10.61%) (Figure 5).

## 4 DISCUSSION

The outcome of our model shows that decreasing connectivity and resource growth select for communities or populations consisting of larger species or individuals due to strong selection for the ability to move over longer distances. Moderate isolation promotes diversity in size, with differently sized individuals able to coexist by foraging at different scales. This increased size diversity also implies higher functional diversity resulting in more efficient resource depletion. Although isolation is the most important driver of consumer body size, resource growth speed is most important for biomass stability, occupancy, and global consumer and resource dynamics. As such, we demonstrate by means of an individual‐based model combining metabolic theory and size‐dependent or size‐independent movement that resource productivity and isolation strongly affect the optimal body size distribution. However, especially at intermediate levels of connectivity, size‐dependent movement is an important driver of body size distributions and the resulting ecological dynamics and functioning.

### 4.1 Isolation and resource growth effects on consumer body size distribution and population dynamics

Our results highlight that consumer body size increases with small‐scale isolation. With increasing isolation, larger individuals are selected as only they are capable of crossing unsuitable matrix to reach neighboring patches. This finding is also supported by other theoretical studies (Etienne & Olff, 2004). However, experimental research has illustrated that habitat fragmentation can have a variable effect on consumer body size within a population or community (e.g., Braschler & Baur, 2016; Davies et al., 2000; Sumner, Moritz, & Shine, 1999). Studies reporting a positive effect contributed this to the positive dependence of mobility on body size (Braschler & Baur, 2016; Jauker et al., 2016; Warzecha et al., 2016). With decreasing growth speed of the resource, the positive effect of isolation on consumer body size is amplified. Logically, as fewer resources are available within the landscape, individuals need to move further to locate them, resulting in stronger selection in favor of a larger body size. The effect of resource growth speed and isolation on the size distribution of the consumer was studied for varying values of the other parameters using a sensitivity analysis (see Supporting Information Appendix S5). Although different‐sized individuals may move at different relative scales, the general trend of increasing body size with isolation and decreasing resource growth speed is always present. With increasing isolation and decreasing resource growth speed, optimal body size increases, thereby amplifying movement distances, but decreasing movement frequency due to lowered local competition. Selection for larger individuals influences population dynamics by resulting in fewer individuals and lower occupancy levels. As such, consumer populations transform from spatially coupled toward classic metapopulations with increasing isolation and decreasing growth speed of the resource (Amarasekare, 2008; Fronhofer, Kubisch, Hilker, Hovestadt, & Poethke, 2012).

At intermediate levels of isolation with moderate or high resource growth speed, the body mass distribution of the consumer has two optima. These optima represent a philopatric and a mobile strategy, which coexist and dominate the population depending on the availability of resources. Our prediction is supported by the observation that both the least and most mobile butterfly species survive best in fragmented landscapes (Thomas, 2000). Our results suggest that when local resource availability is high, the smaller philopatric individuals flourish, while once resources are depleted, only the larger, mobile individuals can trace them within the landscape. The fact that these two strategies coexist while feeding on the same resource by foraging at different scales strongly supports the textural discontinuity hypothesis. This hypothesis states that modes of individual size distributions mirror those scales at which resources within the landscape are most abundant (Borthagaray et al., 2012; Holling, 1992; Laca, Sokolow, Galli, & Cangiano, 2010; Nash et al., 2014). At intermediate levels of isolation, simulations are characterized by the highest diversity in size. Not coincidently, these simulations are also characterized by being most efficient in resource depletion. Because of niche differentiation along the single axis representing “space use”, resources are consumed by each type of body size in a complementing way (Tilman, 2001). As such, populations and communities with a higher diversity in size have a higher functional diversity (Song, Wang, Li, & Zhou, 2014). The observation that size diversity is maximal at intermediate levels of isolation is in line with other studies highlighting a positive effect of fragmentation on species richness (Arnillas et al., 2017; Fahrig, 2017).

Within a spatially implicit model and assuming global dispersal, species diversity is optimized at intermediate dispersal rates, increasing spatial insurance of ecosystem functioning (Loreau, Mouquet, & Gonzalez, 2003). We show in our model that when space is considered explicitly, a high diversity of body sizes is maintained at intermediate isolation. These simulations are also characterized by an average chance of movement. Although higher functional diversity might imply less variability in overall ecosystem functioning when an abiotic condition is fluctuating in space and time (Isbell et al., 2017), this is not the case in our model. We observe no clear link between size diversity and stability in space (*β*_2_ variability) or time at the local (*α* variability) or regional (*γ* variability) scale of consumer biomass. Within our model, no extra abiotic condition is included to which consumers can adapt. Still, resources are heterogeneously distributed in space and might fluctuate in time due to consumption and growth. We allow our consumers to adapt to shifts in resource availability by selection of their size and consequently, their behavior and movement. As such, diversity in consumer size results in optimization of resource consumption at intermediate levels of connectivity but not increased stability of consumer biomass. However, at higher levels of isolation, this positive effect on resource consumption disappears, leading to nonoptimal resource usage at the landscape level. Hence, resource availability is affected, which represents an important ecosystem trait.

The lowest number of consumer individuals occurs when resource growth speed is low, resulting in the least stable population dynamics at all three scales (*α*,* β*_2_, and *γ*). When growth speed is low and isolation intermediate, variability at the regional scale is high, explaining the high number of simulations that went extinct. Surprisingly, *α* and *β*_2_ variability appear to decrease with isolation when growth speed is high. The scenario with both highest isolation and highest growth speed is characterized by high average occupancy but a very low number of individuals, which are large and move rarely but far. This outcome indicates that all these individuals inhabit different cells and are very stationary as resources replenish fast, resulting in the lowest variability of consumer biomass of all scenarios at the local and between‐patch scale. This observation indicates the existence of an important interaction between resource productivity and isolation that should be included when studying ecosystem stability. Still, the stability at the regional scale is unaffected by the level of isolation when growth speed is high.

### 4.2 Importance of size‐dependent movement

Two antagonistic forces regulate metapopulation dynamics: selection in favor of short developmental times that increase net growth rate (acting at the within‐patch scale) and selection in favor of movement (acting at the between‐patch scale) (Davies et al., 2000). Within the coupled model, large individuals are selected when isolation is strong, as only then the benefit of moving far outweighs the disadvantage of developing slowly. However, when decoupling movement speed and body size, an individual's speed of the movement is no longer restricted by its size and instead sampled out of a uniform distribution. As such, the delicate balance between these two forces of selection is disturbed, resulting in generally smaller individuals with fast development rates.

When isolation is high and resource growth speed low, simulations go extinct within the decoupled model as selection for a strategy that guarantees a high movement speed is not possible. Size‐dependent movement is thus essential for the survival of actively moving consumer populations and communities when isolation is strong and resource growth speed low. When isolation is high and growth rate moderate or high, resources are more abundant, which enables populations to persist although average movement speeds are lower than within the coupled model. When immigration of novel genotypes into the metapopulation is allowed, these experience a strong advantage when arriving in unoccupied suitable habitat. As such, they increase migration load and strongly influence the population's average body size, which explains the large amount of variation in average body size within and between simulations. This migration load also explains the high percentage of unexplained variation in weight distribution and corresponding ecological dynamics within the variation partitioning analyses (Table 1). When the immigration of novel genotypes into the metapopulation is deactivated in the decoupled model, smaller body sizes are able to dominate the population or community. Such a decoupling of movement and body size also affects ecological dynamics substantially by resulting in populations and communities with more individuals, which move further and more frequently. Simultaneously, the level of occupancy is increased, which points to spatially coupled populations (Amarasekare, 2008; Fronhofer et al., 2012).

Size‐dependent movement explains most variation in the consumer's body size distribution at intermediate levels of connectivity. This contradicts our expectations as we expected size‐dependent movement to be most essential for the weight distribution of the consumer at the highest levels of isolation. This is also surprising when considering that the effect of decoupling on average consumer body size is largest at high levels of isolation. However, skewness and kurtosis were least affected by the decoupling at these levels of isolation. Also, the largest individuals are selected in scenarios with low growth speed and high isolation, but no comparable simulations could be included of the decoupled model within the analysis as they all went extinct.

The level of isolation has a larger influence on the weight distribution of consumers than the growth speed of the resource or size‐dependent movement. Considering that the weight distribution might be interpreted at the community level with each size class representing a different species, these findings support Watling and Donnelly (2006) who state that the importance of isolation for local species richness is expected to increase with ongoing fragmentation of protected areas.

## 5 CONCLUSIONS

Body size is central to species vulnerability and functioning. The implementation of our mechanistic model enables a deeper and essential understanding of the impact of fragmentation and altered land use on the organization of communities and populations. Here, we demonstrate that size‐dependent movement is vital for the survival of populations experiencing fragmentation by enabling selection for increased movement speed and therefore larger individuals. Also, size‐dependent movement explains most of the observed variation in mass distributions at intermediate levels of connectivity. Further, at these moderate levels of connectivity, local size diversity is highest and hence functional diversity, thereby optimizing resource control but not stability. Moreover, we highlight an important interaction effect between isolation and resource growth on local and spatial variability. Thereby, we contribute to the understanding of the factors that affect body size distributions, enhance size diversity, and thereby indirectly affect the stability and functioning of communities across different scales (Isbell et al., 2017).

## AUTHOR CONTRIBUTIONS

DB, TH, and JH conceived the ideas and designed methodology; JH designed the model; DB, MLV, TH, and JH analyzed the data; and DB, MLV, and JH led the writing of the manuscript.

## DATA ACCESSIBILITY

The applied code is available on https://github.com/jrhillae/coding_manuscript1.
