# Supplementary material for: Size‐dependent movement explains why bigger is better in fragmented landscapes
Source: Ecol Evol. 2018 Oct 23;8(22):10754–67. doi: 10.1002/ece3.4524 (PMC6262741; doi:10.1002/ece3.4524)
Supplement: Supplementary file 1 [file ECE3-8-10754-s001.docx]

# Supplementary material part 1

Table S1.1: An overview of the number of simulations that went extinct

The coupled model

| NND | 0 | 1 | 2 | 3 | 4 | 5 | 6 | 7 | 8 | 9 | 10 |
| --- | --- | --- | --- | --- | --- | --- | --- | --- | --- | --- | --- |
| **r=0.1** | 0 | 0 | 0 | 4 | 8 | 3 | 2 | 0 | 0 | 0 | 3 |
| **r=0.5** | 0 | 0 | 0 | 0 | 0 | 0 | 0 | 0 | 0 | 0 | 0 |
| **r=0.9** | 0 | 0 | 0 | 0 | 0 | 0 | 0 | 0 | 0 | 0 | 0 |

The coupled model- no immigration:

| NND | 0 | 1 | 2 | 3 | 4 | 5 | 6 | 7 | 8 | 9 | 10 |
| --- | --- | --- | --- | --- | --- | --- | --- | --- | --- | --- | --- |
| **r=0.1** | 0 | 0 | 0 | 4 | 7 | 6 | 5 | 4 | 0 | 0 | 10 |
| **r=0.5** | 0 | 0 | 0 | 0 | 0 | 0 | 0 | 0 | 0 | 0 | 10 |
| **r=0.9** | 0 | 0 | 0 | 0 | 0 | 0 | 0 | 0 | 0 | 0 | 0 |

The decoupled model

| NND | 0 | 1 | 2 | 3 | 4 | 5 | 6 | 7 | 8 | 9 | 10 |
| --- | --- | --- | --- | --- | --- | --- | --- | --- | --- | --- | --- |
| **r=0.1** | 0 | 0 | 0 | 0 | 2 | 7 | 10 | 10 | 10 | 10 | 10 |
| **r=0.5** | 0 | 0 | 0 | 0 | 0 | 0 | 0 | 0 | 0 | 0 | 0 |
| **r=0.9** | 0 | 0 | 0 | 0 | 0 | 0 | 0 | 0 | 0 | 0 | 0 |


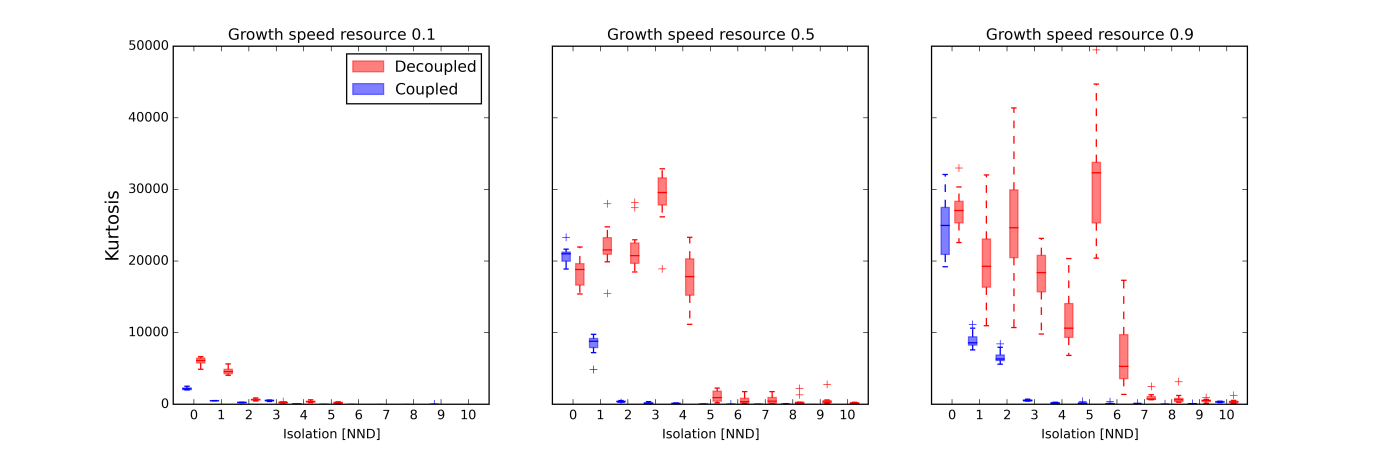


Figure S1.1: The effect of isolation and growth speed on the kurtosis of a consumer’s adult body size (*W_max_*) distribution is displayed. In the coupled model, movement is dependent on body size, while in the decoupled model, both are independent. NND: nearest-neighbor distance expressed in number of cells.
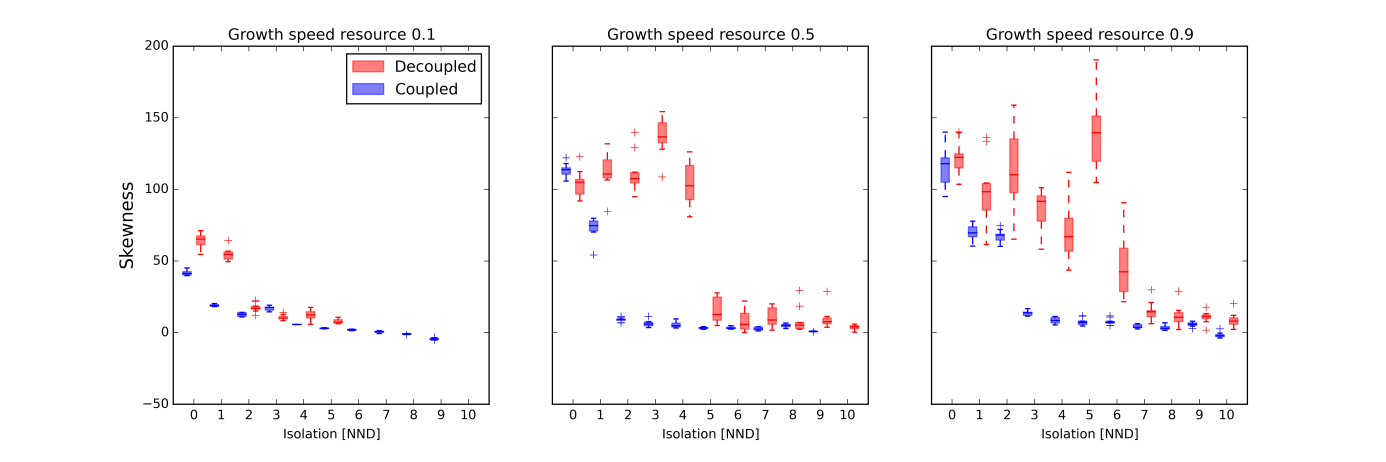
Figure S1.2: The effect of isolation and growth speed on the skewness of a consumer’s adult body size (*W_max_*) distribution is displayed. In the coupled model, movement is dependent on body size, while in the decoupled model, both are independent. NND: nearest-neighbor distance expressed in number of cells.


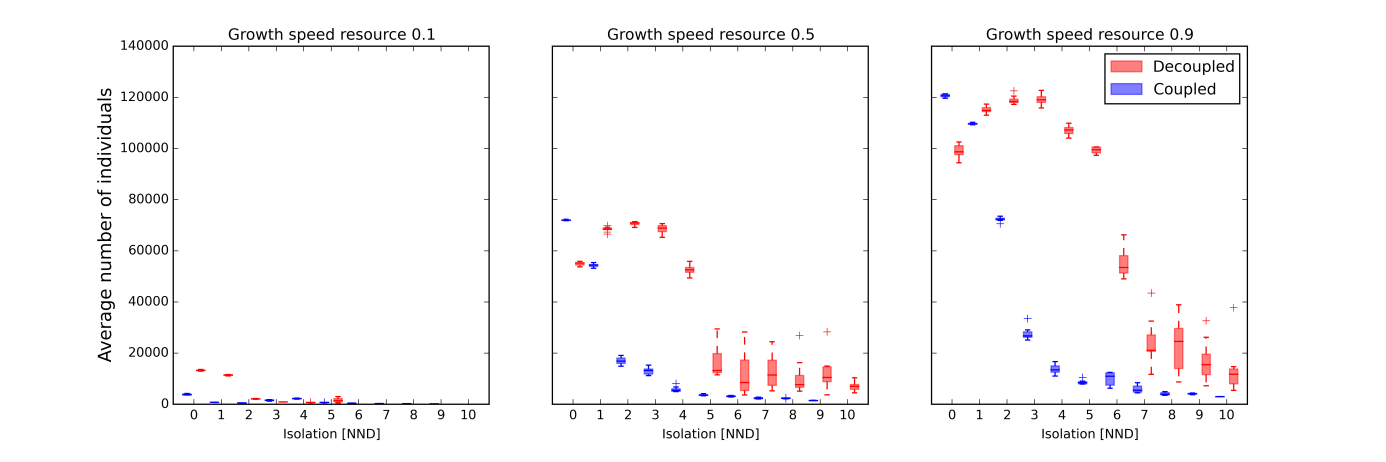


Figure S1.3: The effect of isolation and growth speed on the average total number of consumers is displayed. In the coupled model, movement is dependent on body size, while in the decoupled model, both are independent. NND: nearest-neighbor distance expressed in number of cells.


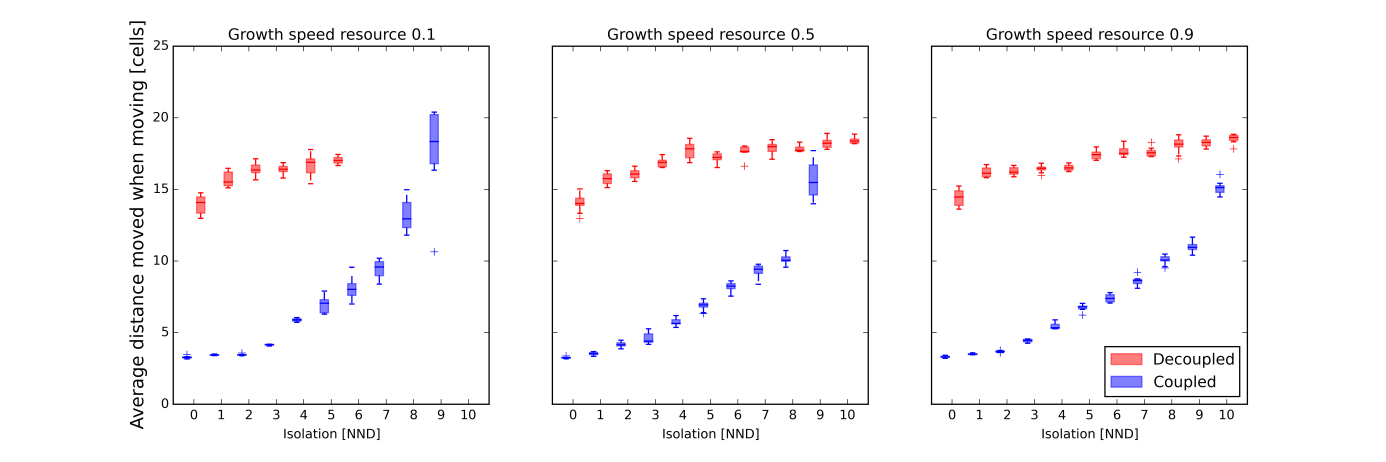


Figure S1.4: The effect of isolation and growth speed on the average distance moved when moving is displayed. In the coupled model, movement is dependent on body size, while in the decoupled model, both are independent. NND: nearest-neighbor distance expressed in number of cells.


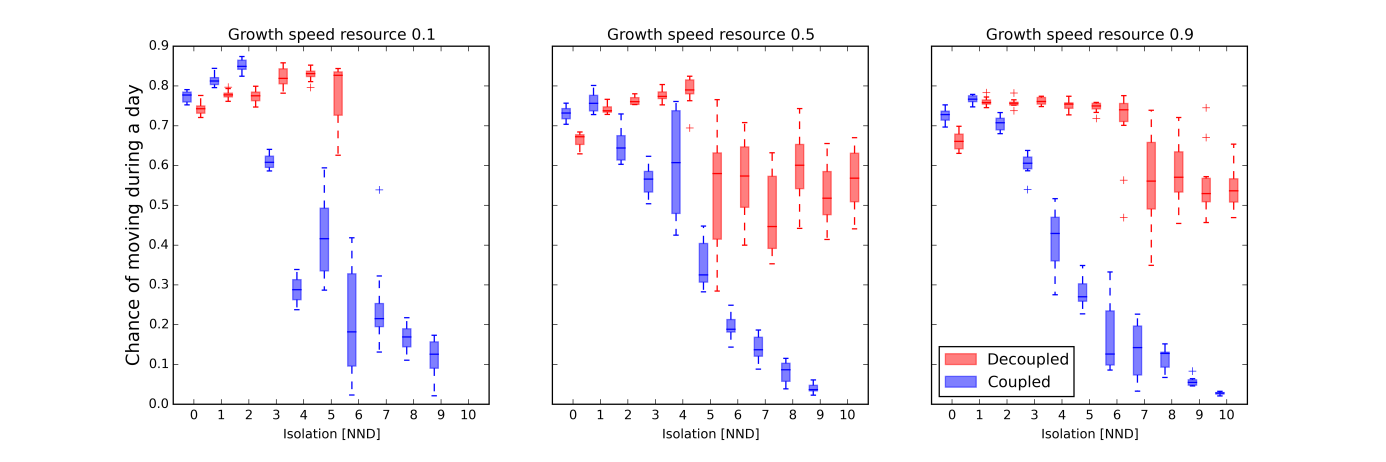


Figure S1.5: The effect of isolation and growth speed on the chance of moving (per day) is displayed. In the coupled model, movement is dependent on body size, while in the decoupled model, both are independent. NND: nearest-neighbor distance expressed in number of cells.


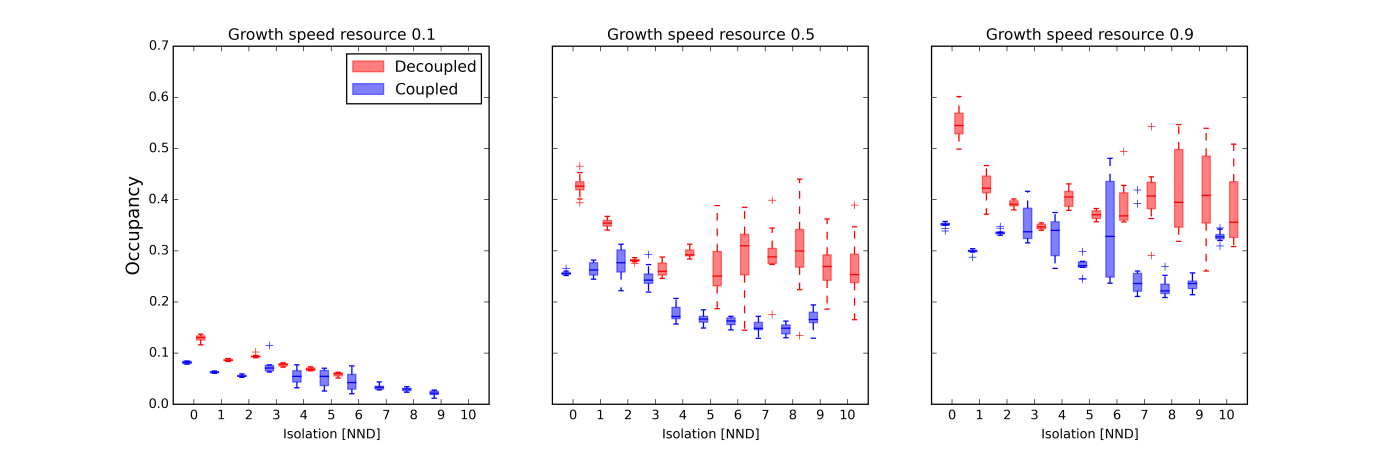
 Figure S1.6: The effect of isolation and growth speed on the relative number of occupied consumer patches is displayed. In the coupled model, movement is dependent on body size, while in the decoupled model, both are independent. NND: nearest-neighbor distance expressed in number of cells.

A
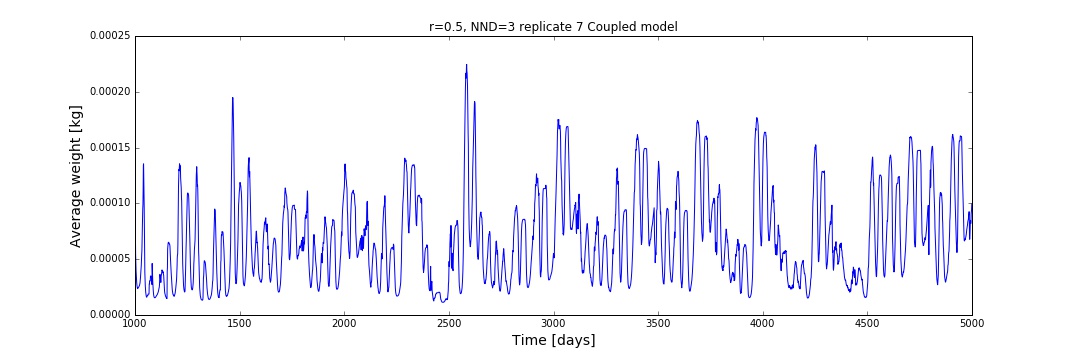


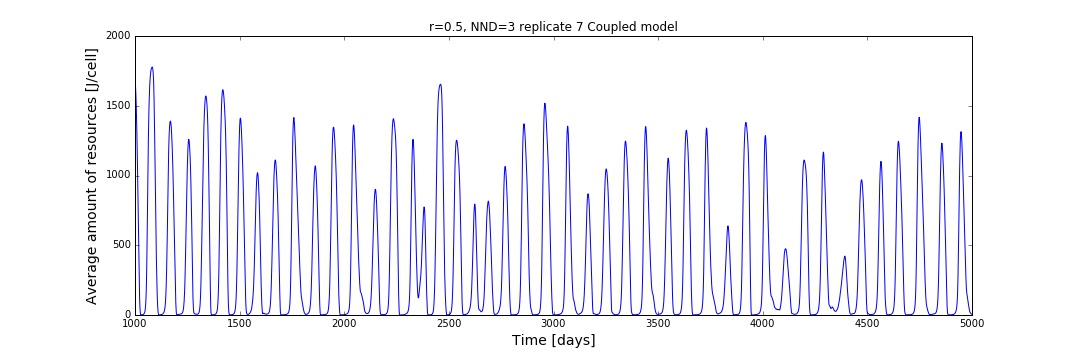


B
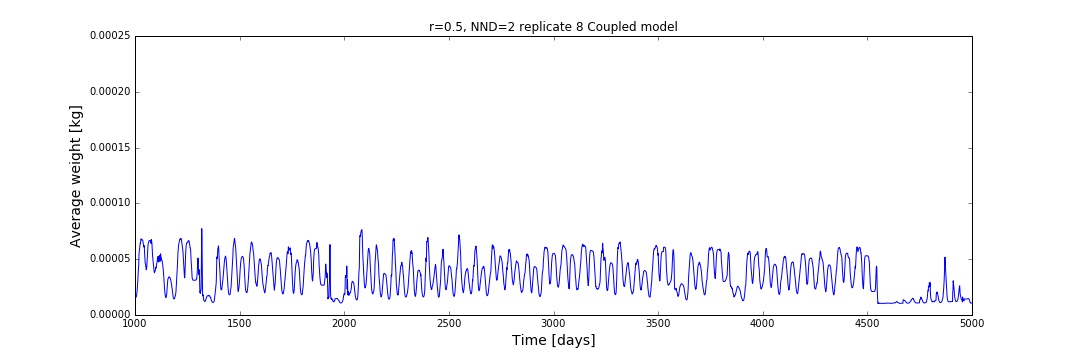

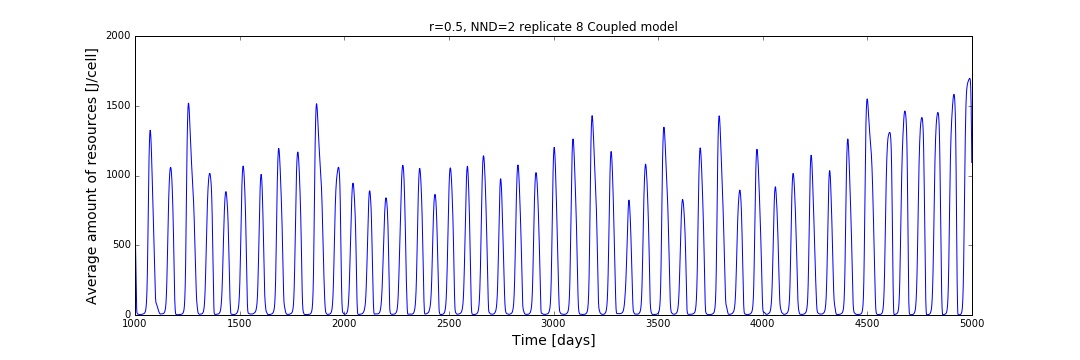
Figure S1.7: Time series illustrating how average body size and amount of resources might fluctuate within the coupled model. A: growth speed 0.5 and NND 3 and B: growth speed 0.5 and NND 2. Please note the difference in average weight between A and B, originating from a difference in level of isolation.


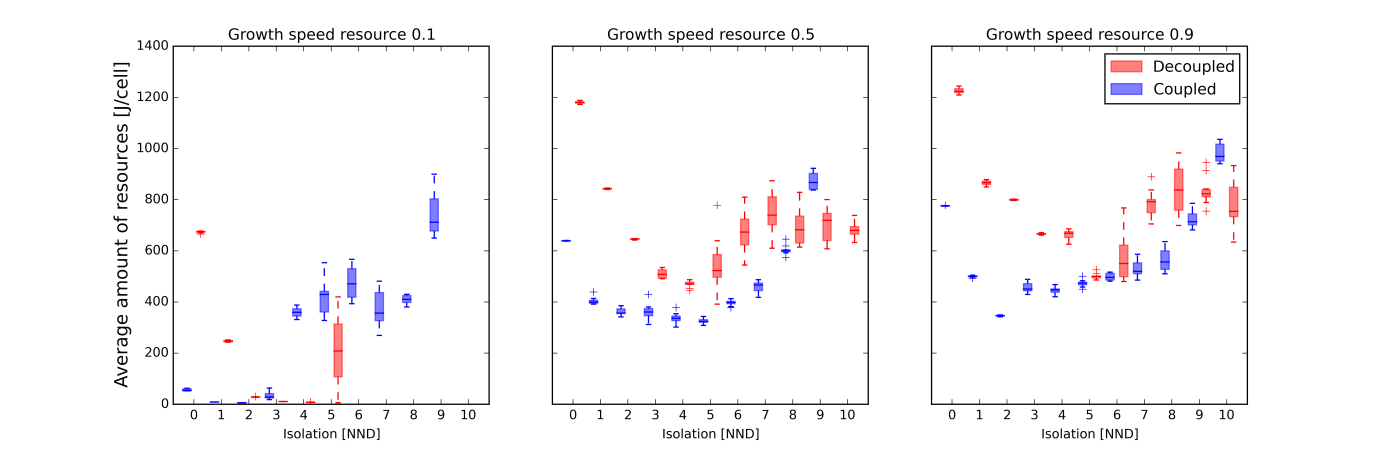
 Figure S1.8: The effect of isolation and growth speed on the average amount of resources present is displayed. In the coupled model, movement is dependent on body size, while in the decoupled model, both are independent. NND: nearest-neighbor distance expressed in number of cells.


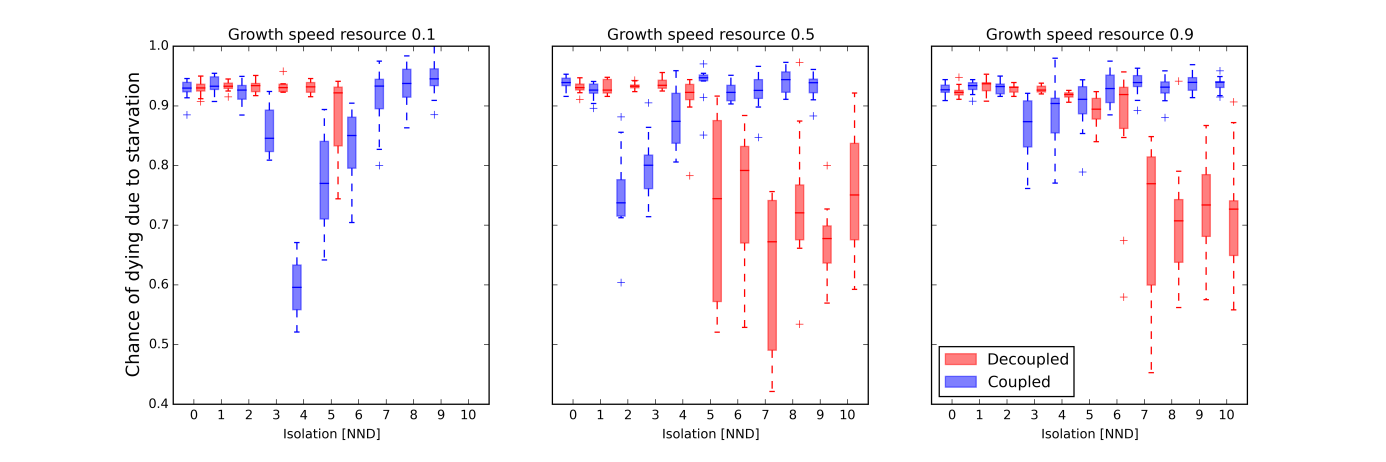
 Figure S1.9: The effect of isolation and growth speed on starvation chance for the consumer is displayed. In the coupled model, movement is dependent on body size, while in the decoupled model, both are independent. NND: nearest-neighbor distance expressed in number of cells.


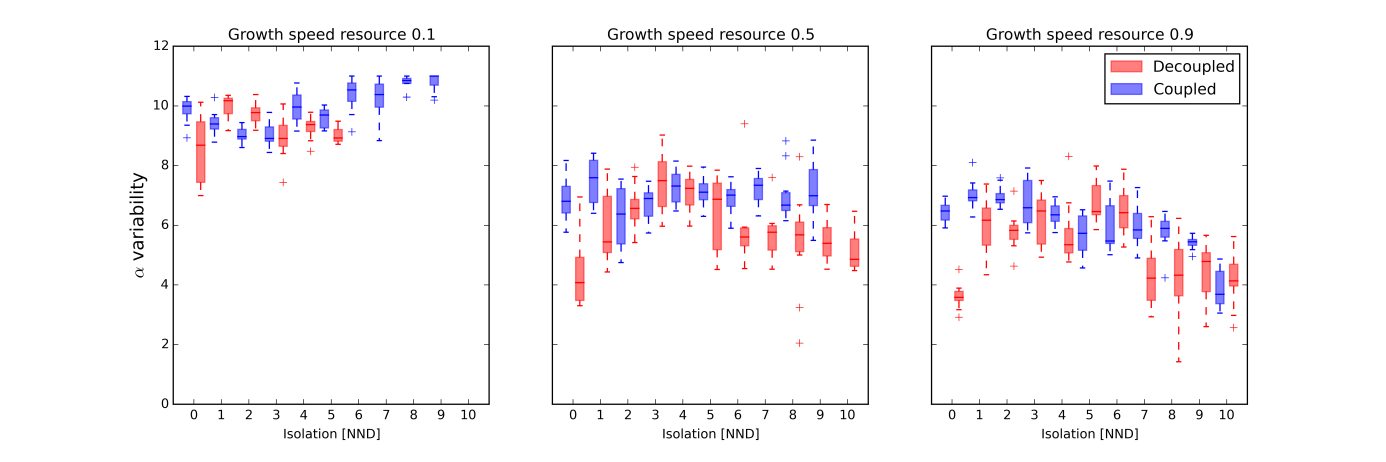
 Figure S1.10: The effect of isolation and growth speed on alpha variability of the consumer’s total biomass is displayed. In the coupled model, movement is dependent on body size, while in the decoupled model, both are independent. NND: nearest-neighbor distance expressed in number of cells.


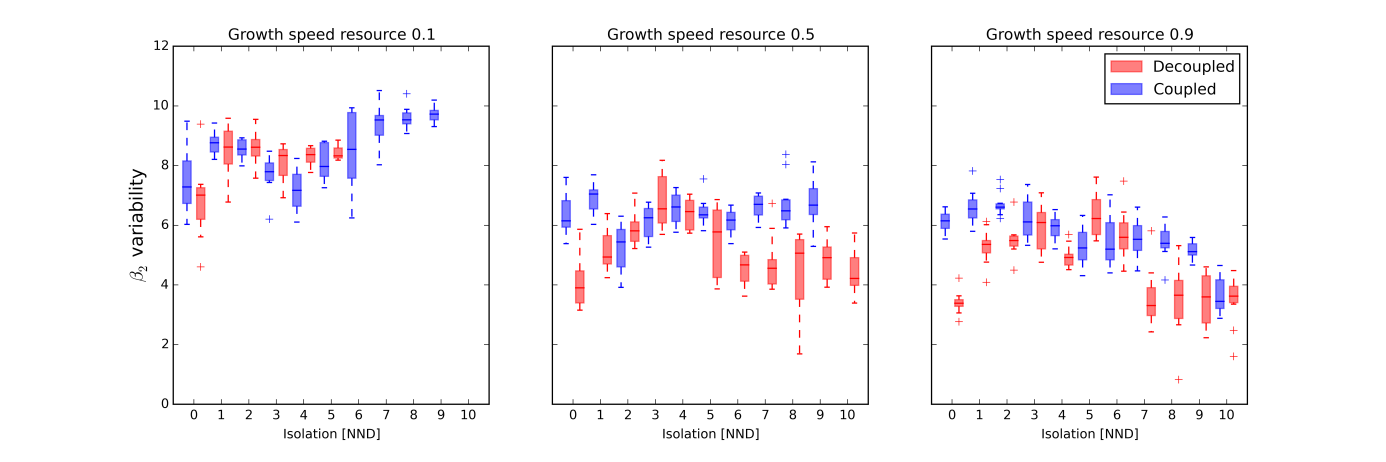
 Figure S1.11: The effect of isolation and growth speed on beta2 variability of the consumer’s total biomass is displayed. In the coupled model, movement is dependent on body size, while in the decoupled model, both are independent. NND: nearest-neighbor distance expressed in number of cells.


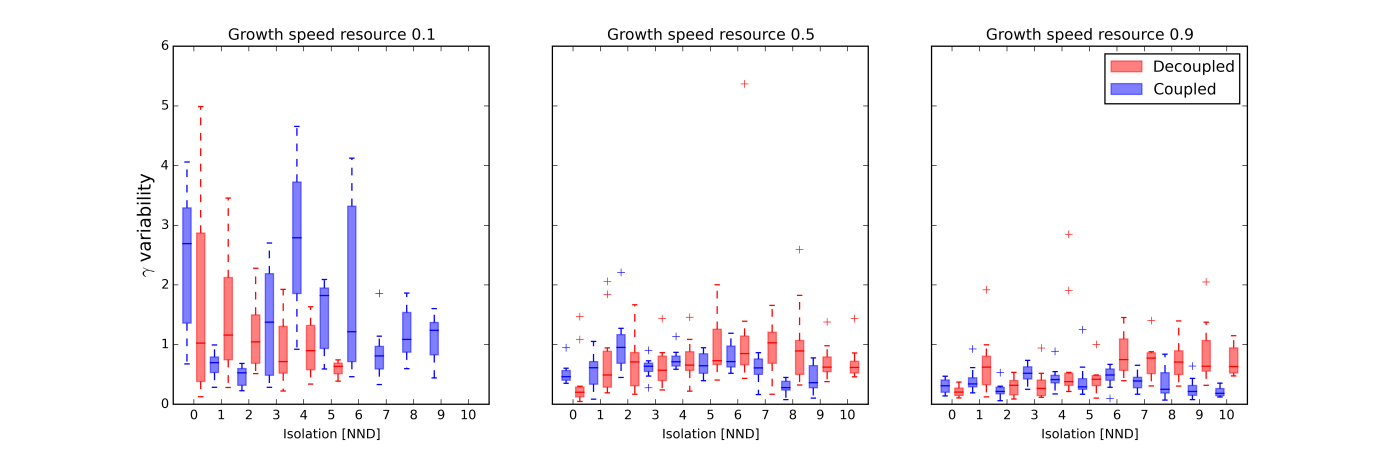


Figure S1.12: The effect of isolation and growth speed on gamma variability of the consumer’s total biomass is displayed. In the coupled model, movement is dependent on body size, while in the decoupled model, both are independent. NND: nearest-neighbor distance expressed in number of cells.


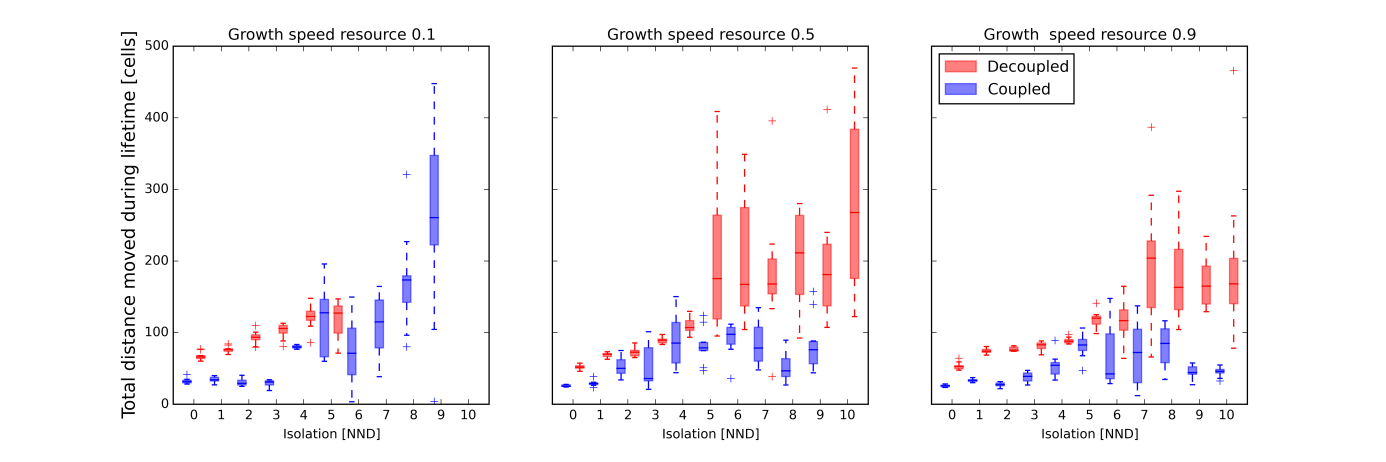
 Figure S1.13: The effect of isolation and growth speed on the total distance moved during a lifetime is displayed. In the coupled model, movement is dependent on body size, while in the decoupled model, both are independent. NND: nearest-neighbor distance expressed in number of cells.


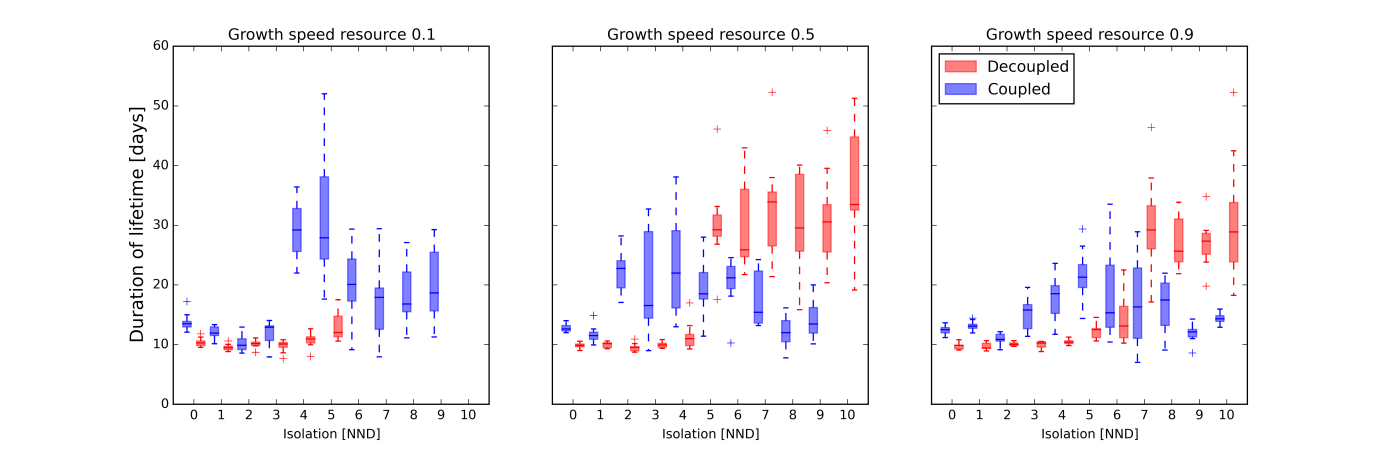
Figure S1.14: The effect of isolation and growth speed on the total lifetime of the consumer species is displayed. In the coupled model, movement is dependent on body size, while in the decoupled model, both are independent. NND: nearest-neighbor distance expressed in number of cells.


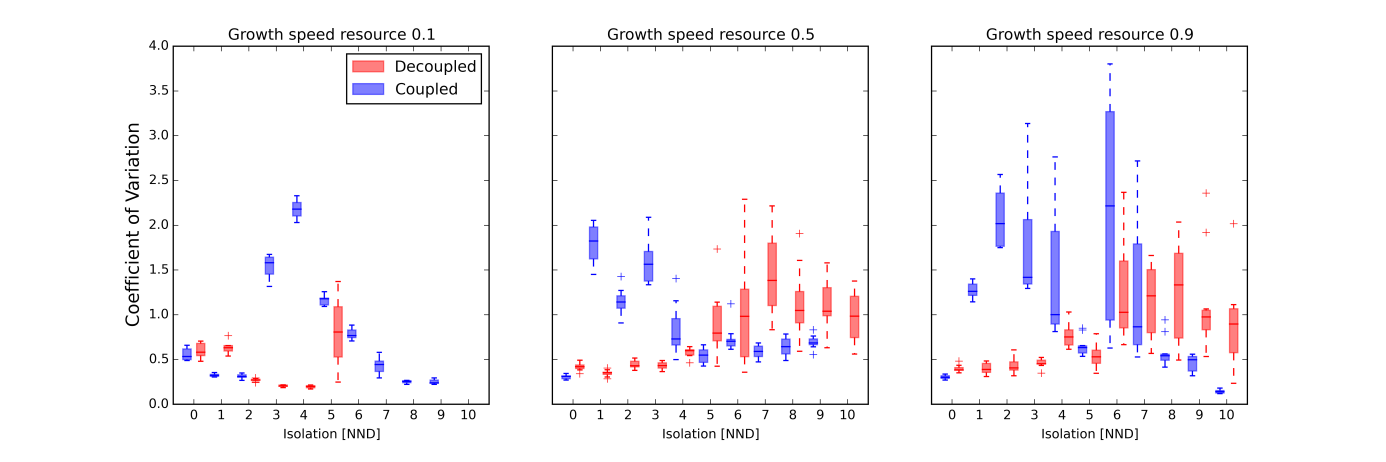
 Figure S1.15: The effect of isolation and growth speed on the coefficient of variation of a consumer’s adult body size (*W_max_*) is displayed. In the coupled model, movement is dependent on body size, while in the decoupled model, both are independent. NND: nearest-neighbor distance expressed in number of cells.


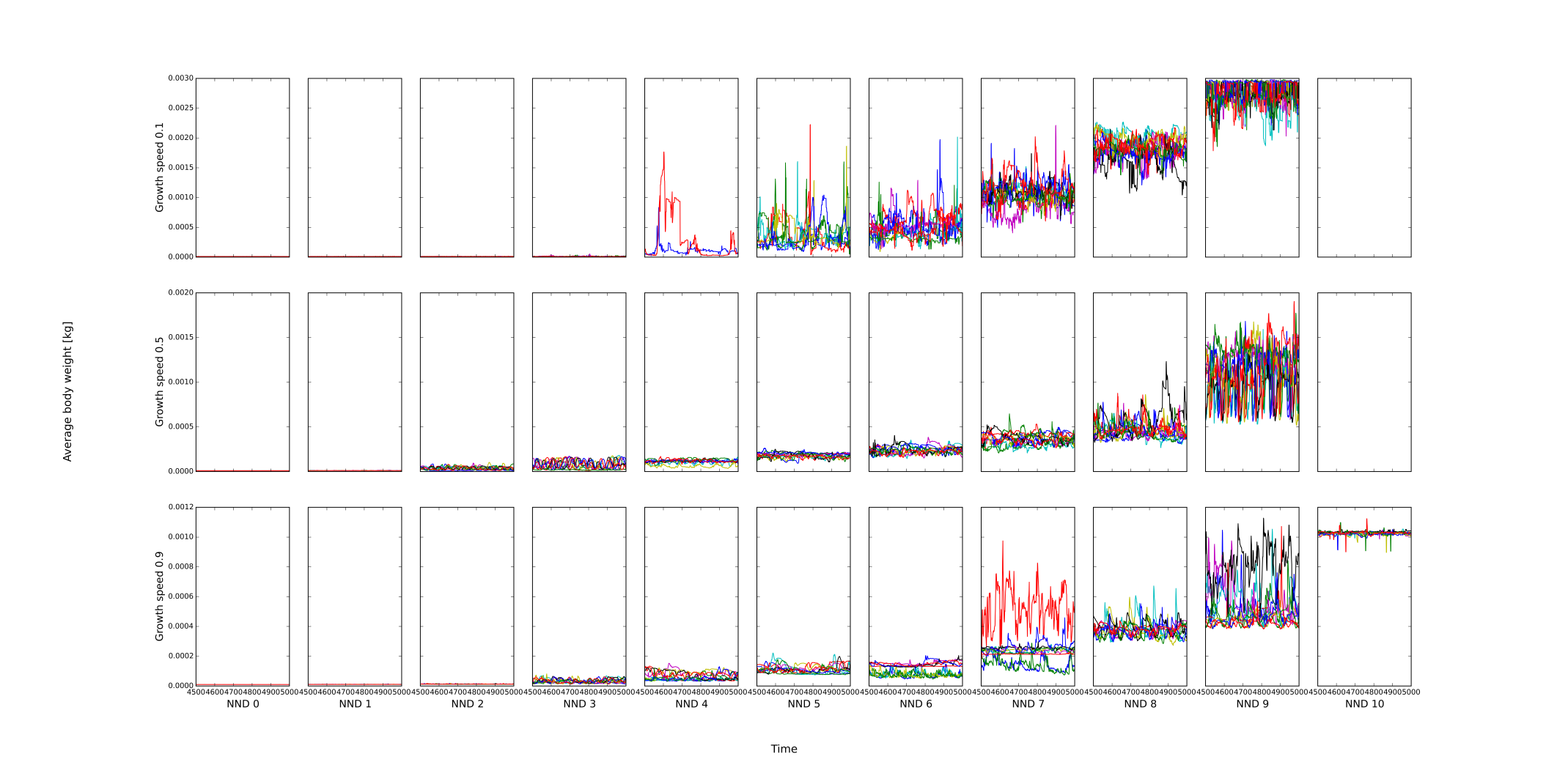


Figure S1.16: A detailed time series of how the average adult body weight of a consumer fluctuates during the final 500 days of a simulation when movement is dependent on size. Simulations differ in degrees of isolation (NND: Nearest Neighbor Distance) and growth speed of the resource.


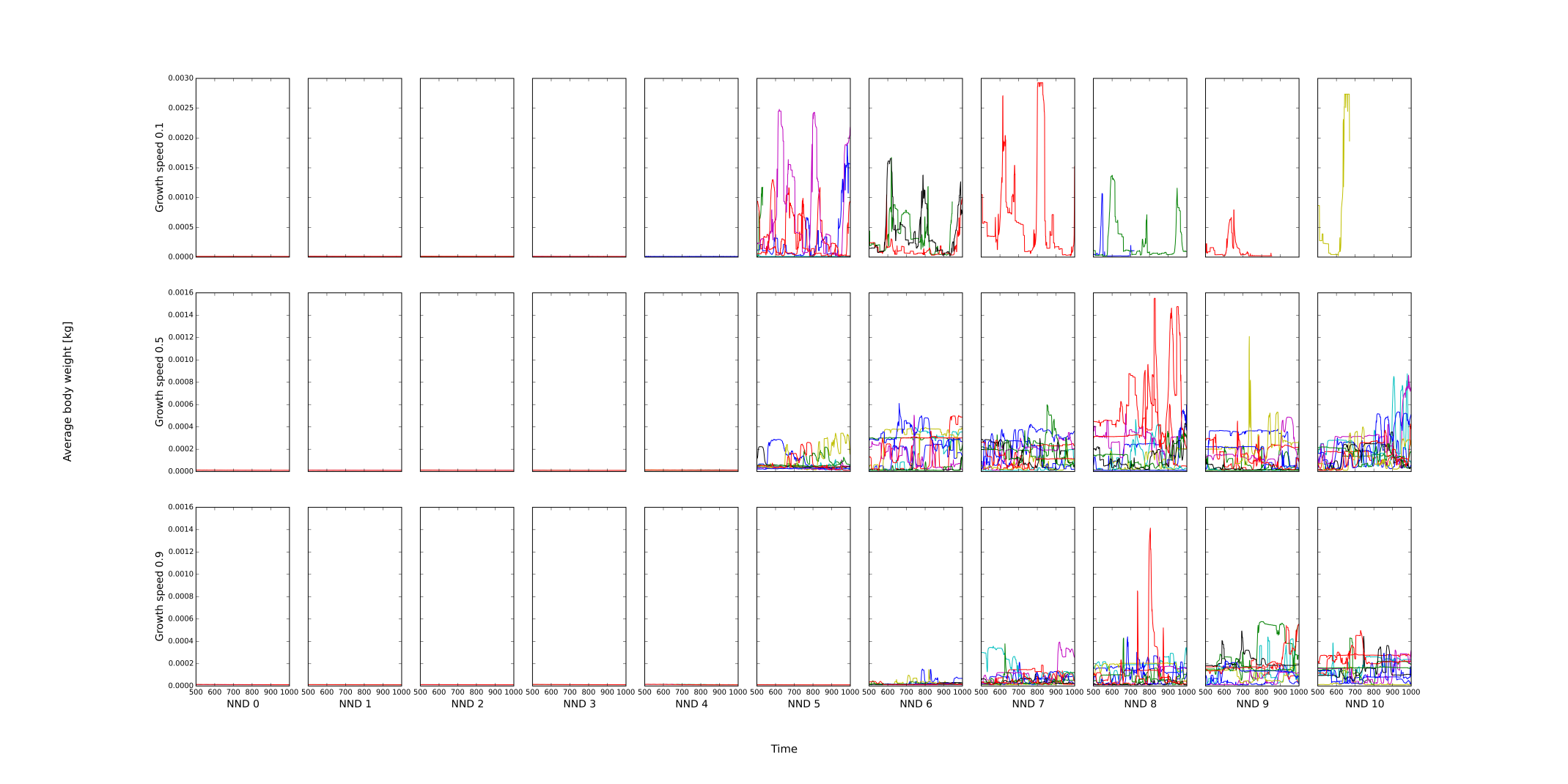


Figure S1.17: A detailed time series of how the average adult body weight of a consumer fluctuates during the final stages of a simulation when movement is independent of size. Simulations differ in degrees of isolation (NND: Nearest Neighbour Distance) and growth speed of the resource.
