# Supplementary material for: Size‐dependent movement explains why bigger is better in fragmented landscapes
Source: Ecol Evol. 2018 Oct 23;8(22):10754–67. doi: 10.1002/ece3.4524 (PMC6262741; doi:10.1002/ece3.4524)
Supplement: Supplementary file 2 [file ECE3-8-10754-s002.docx]

# Supplementary material part 2

While covering a distance *d_max_*, an individual with a perceptual range of *d_per_* will be able to search a total surface of 2× *d_per_* × *d_max_* + *d_per_*^2^× π

*d_per_*

*d_max_*

As each individual has a circular foraging area. The radius of a circle with a surface of 2× *d_per_* × *d_max_* + *d_per_*^2^× π is defined as $\sqrt{\frac{2\times d_{per} \times d_{max} + {d_{per}}^{2}\times\pi}{\pi}}$
