# Supplementary material for: Size‐dependent movement explains why bigger is better in fragmented landscapes
Source: Ecol Evol. 2018 Oct 23;8(22):10754–67. doi: 10.1002/ece3.4524 (PMC6262741; doi:10.1002/ece3.4524)
Supplement: Supplementary file 4 [file ECE3-8-10754-s004.docx]

# Supplementary material part 4

This model description follows the ODD (Overview, Design concepts, Details) protocol for describing individual- and agent-based models (Grimm *et al.*, 2006, 2010).

## 1. Purpose

Our aim was to investigate how spatial isolation and resource growth shape the body size distribution of consumers under scenarios of size-dependent and -independent consumer movement by applying a mechanistic, individual-based resource-consumer model. The outcome was then linked to important ecosystem traits such as resource abundance and consumer stability. Finally, we determined those factors that explain most variation in size distributions.

By applying an individual-based approach, we were able to include intra-specific size variation and stochasticity within our model. This approach in conjunction with the assumption of asexual reproduction and equivalent ontogenetic and interspecific scaling exponents (West, Brown and Enquist, 2001; Moses *et al.*, 2008), implies that our results can be interpreted both at the metapopulation and metacommunity level.

## 2. Entities, state variables, and scales

### The consumer

The consumer species is individually modeled and has the following state variables:

-*W* (kg): referring to the current mass of an individual.

-*W_0_* (kg): the egg mass of an individual

-*W_max_* (kg): referring to an individual’s maximum adult mass.

-*t_d_* (days): referring to the developmental age of an individual.

-*E_r_* (Joule): Each individual has an energy reservoir in which assimilated energy is stored which can be invested in growth, reproduction, movement or the cost of basal metabolic rate.

-Developmental stage: A distinction is made between juvenile and adult consumers. Juveniles invest their assimilated energy in growth, whereas, adults invest their assimilated energy in reproduction.

-Size-dependent or size-independent movement: Within a simulation, size and movement are either dependent or independent.

-x and y coordinate

### The landscape

The configuration of the landscape is defined by two state variables:

-*NND*: Nearest neighbor distance, describing level of isolation

-*r*: growth speed of the resource within a suitable cell

The landscape is cellular consisting out of several grid cells.

### A grid cell

The resource species is not individually modeled, instead, resource amounts are defined per cell.

Per cell, the following state variables are defined:

-Suitability: Whether or not a cell is suitable for the resource to grow.

- *R_x,y_* (Joule): Per cell a resource amount is defined in Joule.

-*K* (Joule): Carrying capacity of the resource (Joule).

-*r*: Growth speed of the resource (Joule).

-*Enc* (Joule): amount of resource present per cell which is non consumable by the consumer.

-A list of consumers present in a particular cell

-*p* : probability of moving of the consumer is calculated per time step per cell

- x and y coordinate

### Scales

All landscapes have a constant number of suitable patches (i.e. 2500) but varying nearest neighbor distance (*NND*) (Fahrig, 2003). The effect of isolation is tested by assigning a constant *NND* from 0 to 10 to all cells (see supplementary material part 3 for an example). Consequently, the dimensions of the landscape increase with *NND* according to (50 + *NND**50) × (50 + *NND**50) cells. The boundaries of the landscape are wrapped. Each cell has a dimension of 0.25×0.25m. One time step corresponds to one day. For the coupled model, all simulations ran for 5000 time steps. Because of computational limitations, the number of time steps of the decoupled model differed between simulations (see Table S4.2). If the dynamics allowed for the existence of an equilibrium, this was obtained (see Figure S1.16 & S1.17).

## 3. Process overview and scheduling


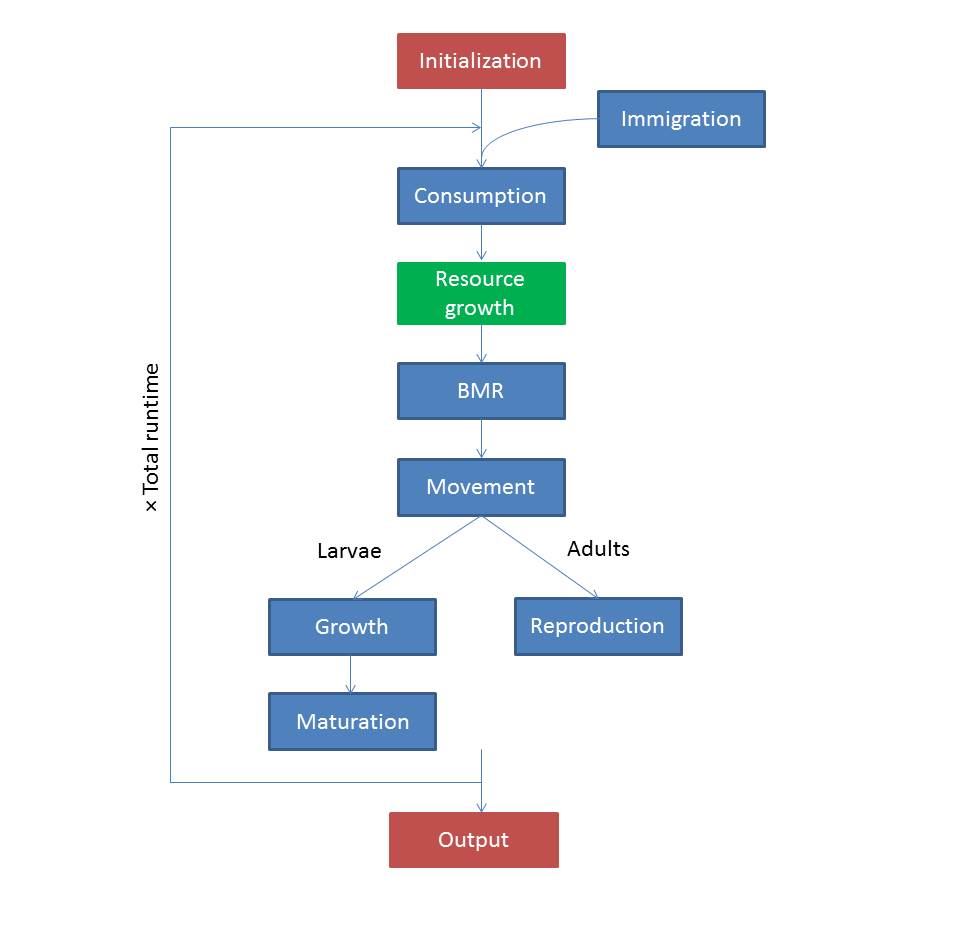


Figure S4.1: This figure depicts an overview of all events within the model. BMR stands for the basal metabolic rate costs. Initialization and output generation are regulated at the level of the model. Blue boxes do represent events of the consumer and the green box represents the event regulating resource growth.

The applied model is a spatially explicit, discrete-time model with overlapping generations. One time step corresponds to one day within the lifetime of the consumer. We here took an arthropod-centered approach and parameterized allometric rules for a haploid, parthenogenetic arthropod species feeding on plants (the resource), with a semelparous lifecycle.

In case immigration is allowed (according to immigration rate *q*), an immigrant is initialized within the landscape at the start of a day.

Next, an individual nourishes its energy reserve by consumption. Second, the energy reserve is depleted by the cost of daily maintenance (i.e. basal metabolic rate) and the cost of movement. Third, juveniles may further deplete the energy reserve by growth, eventually resulting in maturation if they approximate their adult mass (*W_max_*). Consumed energy that was not expended during a day remains in the energy reserve. Adults can only reproduce if their internally stored energy (*E_r_*) exceeds a predefined amount. As the consumer species is semelparous, adults die after reproduction.

During growth, an individual’s mass is updated. When moving, an individual’s x and y coordinate change. If maturation is allowed, a larva becomes an adult (updating an individual’s developmental stage). Resource quantities per cell decrease during consumption but replenish during resource growth.

All events are executed simultaneously by all consumers. As competition for resources is scramble, the order at which individuals consume does not affect the outcome.

During a simulation, summarizing output variables are collected, which are written out at the end of a simulation.

## 4. Design concepts

Basic principles:
An organism’s body size is one of its most comprehensive characteristics. As described by the metabolic theory of ecology, body size is strongly correlated with an array of functional traits (Peters, 1983; Brown *et al.*, 2004). Based on this theory, we linked many individual traits to body size (e.g. basal metabolic rate, movement speed, ingestion rate, clutch size, developmental time). It is crucial to include these allometric rules within the model as body size represents the outcome of several selective pressures acting on different life history traits, setting boundaries to the ecology, physiology and functioning of an individual (Peters, 1983).
As habitat fragmentation and destruction progress, the spatial distribution of resources is altered. The importance of resource distribution for body size distributions is advocated by the ‘textural discontinuity hypothesis’ of Holling (Holling, 1992; Gagné, Proulx and Fahrig, 2008; Raffaelli *et al.*, 2016). We test this hypothesis by investigating the effect of isolation and resource growth on body size distributions. The importance of the allometric rule linking body size and movement is illustrated by comparing the outcome of two different models: a size-dependent movement model and a size-independent movement model. Finally, important ecosystem traits such as resource availability and consumer stability are also studied.

Emergence:

As habitat fragmentation and destruction progress, the spatial distribution of resources is altered, yet the consequences for (future) body size distributions are unclear. On the one hand, large-bodied individuals may be selected as they have high starvation resistance and are able to cover large distances (Peters, 1983; Davies, Margules and Lawrence, 2000; Tscharntke and Brandl, 2004). On the other hand, small-sized individuals may have the benefit of short developmental times and low energy requirements (Peters, 1983). Therefore, the most import output of the model are the observed differences in body mass distributions between simulation with different levels of isolation and resource growth. Moreover, we do determine how strongly the outcome of the coupled model depends on the assumption of size-dependent movement.
Body size distributions will consequently feedback on the overall food web dynamics (overall instability and resource availability). These feedbacks are also investigated by collecting data of fluctuations in resource abundance and consumer numbers.

### Adaptation:

Body size is linked to many features of an individual. In this model, larger individuals move faster, develop longer, have larger clutch sizes, have larger basal metabolic rates, and have higher ingestion rates. These traits also change during the developmental phase of an individual, corresponding to its body mass.

Further, the energy invested in growth and movement depends on the energy available within an individual’s energy reserve. As such, an individual will never die due to exhaustion by moving too far. Also, the available energy is invested in the events in the following order: (1) basal metabolic rate, (2) movement and (3) growth or reproduction. As such, an individual’s priority is investing energy in basal metabolic rate cost. Secondly, it will try to guarantee access to resources the following day by moving. Thirdly, it will invest remaining energy in growth or reproduction if some energy is left. Energy for reproduction is collected during several days as only one clutch is produced during the lifetime of an individual.

Probability of moving (*p*) decreases with resource availability in a cell but also increases with the sum of the ingestion rates of all individuals within that cell. As such, individuals have higher chances of moving when a lot or large conspecifics are present and resources are scarce.

### Objectives:

The short term objective of an individual, is to consume resources. The long term objective, however, is to produce a clutch of individuals. Those individuals which have the most optimal body size, are selected and as such most abundant within the community or population.

Sensing:

When present within a cell, an individual may sense the amount of resources present and also estimate the total consumption pressure within that cell. Based on this information it decides whether it will start moving or not. When moving, an individual can sense the exact amount of resources within a cell.

### Interaction:

Interactions between individuals are always indirect. During consumption, competition for resources is scramble. Also, the number and size of consumers present within a cell are taken in account when calculating the probability of moving for individuals within that cell.

### Stochasticity:

Stochasticity is included when defining which individuals will move from a particular cell. Also, stochasticity regulates the mutation events of the allele describing the maximum mass of an individual.

When multiple cells exist with the maximum amount of resources within an individual’s searching area during movement, stochasticity is also implemented.

### Collectives:

Between consumer individuals, a distinction is made between juveniles and adults. Juveniles invest their energy in growth, whereas, adults invest their energy in reproduction.

### Observation:

During each simulation, we traced changes in the mean amount of resources per cell, total number of adults and juveniles, average adult mass (*W_max_*) and the coefficient of variation, skewness, and kurtosis of the consumer’s adult mass (*W_max_*) distribution. Every 500 time steps, the value of *W_max_* of maximum 50 000 randomly sampled individuals was collected.

#### Occupancy (O)

Occupancy (*O*) is defined as the ratio of occupied patches to the total number of suitable patches within the landscape. The level of occupancy is determined every ten days during the last 100 days of a simulation. At the end, the average of these values is calculated per simulation.

#### Variability

In order to infer the temporal stability of the community at different scales we calculated the *α*, *β_2_* and *γ* variability for each simulation run. This calculation is based on samples of total consumer biomass every 10 time steps during the final 100 time steps of a simulation within 100 pre-selected, suitable cells. *α* variability is a measure of the local temporal variability and is calculated by

$\alpha_{\mathrm{CV}}=\left( \frac{\sum_{m} \sqrt{w_{m}}}{\sum_{m} \mu_{m}} \right)^{2}$ $\alpha_{\mathrm{CV}}=\left( \frac{\sum_{m} \sqrt{w_{m}}}{\sum_{m} \mu_{m}} \right)^{2}$ (eq. 1)

with *w_m_* referring to the temporal variance and *µ_m_* to the temporal mean of population or community consumer biomass in cell *m* (Wang and Loreau, 2014). The temporal variability at the metapopulation or metacommunity scale or *γ* variability was calculated by:

$\gamma_{CV}=\frac{\sum_{m,n} w_{mn}}{\left( \sum_{m} \mu_{m} \right)^{2}}$ (eq.2)

with *w_mn_* referring to the temporal covariance of population or community biomass between cells *m* and *n* (Wang and Loreau, 2014). Finally, *β_2_* variability or asynchrony-related spatial variability was determined by:

$\beta_{2}=\alpha_{CV}-\gamma_{CV}$ (eq. 3).

#### Reproductive success and movement

Throughout the final 600 days of a simulation, 1000 eggs were randomly selected to be followed during their lifetime. The movements and reproductive success of the resulting consumer individuals were recorded.

#### Variation partitioning

By means of multivariate variation partitioning we disentangled the amount of variation in adult size that can be explained by the coupling of body size and movement, resource growth rate and level of isolation. Analysis were performed in R by applying the function varpart within the package vegan which is based on calculating the adjusted *R^2^* in redundancy analysis ordination (RDA) (Oksanen *et al.*, 2018). This was done by collecting the average, coefficient of variation, level of skewness, and level of kurtosis of the distribution of *W_max_* per simulation. We also executed a similar analysis for (i) occupancy, (ii) parameters summarizing resource and consumer dynamics (resource abundance, resource variance and consumer abundance) and (iii) the metapopulation functioning statistics *α*, *β_2_,* and *γ* variability. We executed a global variation partitioning including all distances except for *NND* 10 as some of these simulations were not stable and only survived as sinks. We furthermore executed a variation partitioning for each value of *NND* independently. As such, the effect of isolation on the amount of variation explained by the coupling of body size and movement could be estimated. In order to guarantee that each parameter contributed equally, all data were *z* transformed prior to analysis.

## 5. Initialization

Per parameter combination, 10 simulations were run. At the start of each simulation, 1000 adult individuals were placed into the landscape. The adult mass of each individual (*W_max_*) was defined as ten raised to the power of a value drawn from the uniform interval [-5, -2.522878745]. In other words, we sample a value between 0.00001 kg (minimum adult mass) and 0.003 kg (maximum adult mass). As such, individuals with masses of different orders of magnitude have an equal chance of being initialized in the landscape. Moreover, initialized distributions are skewed to small individuals. Also, each initialized individual carried enough energy within its energy reserve to survive the first day. This amount of energy is calculated based on an individual’s mass and accounts for the cost of basal metabolic rate and movement during one day. Initial resource availability per cell corresponded to the maximum carrying capacity.

## 6. Submodels

### The resource

As it is advisable not to focus on individual species but also cover their interactions with other species (Berg *et al.*, 2010), we included the dependence of the consumer on its resource by varying the resource’s growth speed. Resources at the cell level are not individually modeled but by a local logistic growth model. Local resource biomass is represented as the total energetic content of resource tissue within that cell (*R_x,y_* in joules). This resource grows logistically in time depending on the resource’s carrying capacity (*K*) and intrinsic growth rate (*r*). *K* was set to 2000 *J* [assumption of space limitation] whereas *r* differed between simulations (0.1, 0.5 or 0.9 per day; assumption for productivity of the system). In any cell, a fixed amount of resource tissue (*E_nc_*, in Joules, fixed at *1* *J*) is non-consumable by the consumer species, representing below-ground plant parts. As such, *E_nc_* is the minimum amount of resource tissue present within a suitable cell, even following local depletion by the consumer species.

### The consumer

All consumers are individually modelled within the landscape. The consumer has two life stages: a juvenile and adult life stage. Within a day, both stages have the chance to execute different events (see Figure 1).

First, an individual nourishes its energy reserve by consumption. Second, the energy reserve is depleted by the cost of daily maintenance (i.e. basal metabolic rate). Third, an individual has the opportunity to move. Fourth, juveniles may grow, eventually resulting in maturation if they approximate their adult mass (*W_max_*). Energy for reproduction is collected during several days as only one clutch is produced during the lifetime of an individual. The energetic threshold for reproduction increases with body size.

Moreover, the energy of an individual’s energy reserve (*E_r_*) is invested in the following order: (1) basal metabolic rate, (2) movement and (3) growth or reproduction. As such, an individual’s priority is investing energy in basal metabolic rate cost. Secondly, it will try to guarantee access to resources the following day by moving. Thirdly, it will invest remaining energy in growth or reproduction if some energy is left. As the consumer species is semelparous, adults die after reproduction.

Energy from consumed resources that was not expended during a day remains in the energy reserve. Body size is linked to many features of an individual. In this model, larger individuals move faster, have longer developmental times, larger clutch sizes, higher basal metabolic rates, and higher ingestion rates. These traits also change during the developmental phase of an individual, corresponding to its body mass.

Individual body mass at maturity (*W_max_*, in kg) is coded by a single gene. Adult mass is heritable and may mutate with a probability of 0.001 during reproduction. This mutation rate is commonly applied within theoretical models (Travis *et al.*, 2009; Henry, Coulon and Travis, 2015). A new mutation is drawn from the uniform distribution [*W_max_* – (*W_max_*/2), *W_max_* + (*W_max_*/2)] with *W_max_* referring to the adult mass of the parent. New mutations may not exceed the predefined boundaries [0.01g, 3g] that represent absolute physiological limits. As such, our minimum adult mass corresponds to the mass of a small grasshopper such as *Tetrix undulata* (0.01 g) and the maximum mass (3 g) to that of some longhorn beetles (Cerambycidae), darkling beetles (Tenebrionidae), scarab beetles (Scarabaeidae) or grasshoppers (Acrididae). New variants of this trait may also originate by immigration (see below). Mutation enables fine-tuning of the optimal body size, whereas immigration facilitates fitness peak shifts.

### The landscape

The landscape is cell-based with each cell having a side length (*SL*) of 0.25 m. This fine-grained fragmentation is relevant for studying the effect of isolation on the foraging behavior of arthropods.
Within the landscape, a distinction is made between suitable and unsuitable habitat. Resources only grow within suitable patches with one patch having the size of a single cell. All landscapes have a constant number of suitable patches (i.e. 2500) but varying nearest neighbor distance (*NND*) (Fahrig, 2003). The effect of isolation is tested by assigning a constant *NND* from 0 to 10 to all cells (see supplementary material part 3 for an example). Consequently, the dimensions of the landscape increase with *NND* according to (50 + *NND**50) × (50 + *NND**50) cells. The boundaries of the landscape are wrapped.

#### Immigration

The frequency with which immigrants arrive in the landscape is described by *q*. This variable is fixed at one per 10 days. The process of determining an immigrant’s adults mass is similar as during initialization. An immigrant is always introduced within a suitable cell and its energy reserve contains just enough energy to survive the first day. This amount of energy is calculated based on an individual’s mass and accounts for the cost of basal metabolic rate and movement during one day.

#### Consumption

Individual ingestion rate (*IR*, in Watts) of an individual increases with its mass (*W*, in kg) by the following equation:

$IR=2*W^{0.80}$ (eq. 4).

Following log transformation, the slope (0.80) was found by Peters (1983) to be the mean of several studies focusing on ingestion rates of poikilotherms (Peters, 1983). The intercept of this equation lays within the observed range of elevations [0.12 to 2] of these studies (Peters, 1983).

Based on eq. 4, the amount of energy ingested per day for an individual (*i_max_* in Joules) is determined as

$i_{max}=2\cdot W^{0.80}\cdot t_{f}$ (eq. 5)

with *t_f_* referring to the time devoted per day to consumption (in seconds), which is fixed at 15 hours. Individuals compete for resources by means of scramble competition according to the following equation:

$E_{c}=i_{max}$ if $\sum{i_{max}}_{x,y}\leq$ $R_{x,y}$ (eq. 6)

$E_{c}=R_{x,y}\cdot\frac{i_{max}}{\sum{i_{max}}_{x,y}}$ if $\sum{i_{max}}_{x,y}>$ $R_{x,y}$

with *E_c_* being the energy consumed by an individual, *R_x,y_* the total amount of energy available within cell*_x,y_* and $\sum{i_{max}}_{x,y}$ the sum of the ingestion rates of all individuals present within cell*_x,y_*. However, when we consider that the consumer feeds on young terrestrial foliage, it may only assimilate 65 percent of its daily ingested energy (Ricklefs, 1974 cited in Peters, 1983). Moreover, we assume that the consumer loses 10 percent of its ingested energy to processing costs (i.e. specific dynamic action) (Ricklefs, 1974 cited in Peters, 1983). As such, only 55 percent of the ingested energy remains available to the organism. Therefore, the energy that is being assimilated by an individual (*E_a_* in Joules) is described by

$E_{a}=0.55\cdot E_{c}$ (eq. 7)

#### Basal metabolic rate

The standard metabolic rate of poikilotherms (*M*, in watts) is described by

$M=0.14W^{0.751}$ (eq. 8)

with *W* being body mass (in kg) (Hemmingsen, 1960 cited in Peters, 1983). As such, the total standard metabolic rate costs per day (*M_day_*) are calculated according to

$M_{day}=0.14W^{0.751}*\left( 3600*24-t_{m} \right)$ (eq. 9)

If an individual has stored less energy within its reserve (*E_r_*) than *M_day_*, it dies at the beginning of the day. The standard metabolic rate exhibited during movement is already included within the cost of movement (Peters, 1983). Therefore, the cost of daily existence is only calculated based on the time that an individual is not moving per day. The time an individual moves per day (*t_m_*, in seconds) varies between individuals and over time (see below).

#### The movement phase

##### **Probability of moving (*p*)**

Whether an individual moves depends on the ratio of the amount of energy present within a cell $\left( R_{x,y} \right)$relative to the maximum amount of energy that can be consumed by all consumers present within that cell. This latter factor is determined by calculating the sum of all individuals’ daily ingestion rates within that cell ($\begin{aligned} \begin{aligned} \begin{aligned} \begin{aligned} \sum{i_{max}}_{x,y} \end{aligned} \end{aligned} \end{aligned} \end{aligned}$).
By assuming a symmetric competition, the probability of moving (*p*) is equal for all individuals present within the same cell and is calculated by (based on (Poethke and Hovestadt, 2002)) :

$p=1-\frac{R_{x,y}}{\sum{i_{max}}_{x,y}}$ if $\frac{R_{x,y}}{\sum{i_{max}}_{x,y}}<1$ (eq. 10)

$p=0$ if $\frac{R_{x,y}}{\sum{i_{max}}_{x,y}}\geq1$

##### Defining searching area

As one time step in our model corresponds to one day, we do not model the movement behavior of an individual explicitly but instead, estimate the total area an individual can search for resources during a day. This area is called an individual’s searching area is calculated once per time step, for each moving individual. As all cells at a particular distance from the origin are equally intensively searched, the searching area is circular with a radius (*rad*) and a center corresponding to the current location of an individual (Delgado *et al.*, 2014). An individual’s searching area increases with an individual’s optimal speed (*v_opt_*), movement time (*t_m_*) and perceptual range (*d_per_*). Both optimal speed and perceptual range depend on body mass, resulting in larger searching areas for larger individuals. The cost of movement includes the energy invested by an individual in prospecting its total searching area. Therefore, it is dependent on the size of the total searching area instead of the shortest distance between the cell of origin and cell of destination.

An individual’s average speed of movement (*v_opt_*, in meters per second) is calculated by means of the following allometric equation, derived for walking insects ( Buddenbrock, 1934 cited in Peters, 1983):

$v_{opt}=0.3\cdot W^{0.29}$ (eq. 11).

With *W* referring to the mass of an individual in kg, while ignoring the mass of stored resources. The time an individual invests in movement per day (*t_m_*, in seconds) is maximally 1 hour. In case too little internally stored energy (*E_r_*) is present to support movement for one hour, *t_m_* is calculated by:

$t_{m}=\frac{E_{r}}{c_{m}}$ (eq. 12).

*c_m_* refers to the energetic cost of movement (in joules per second) and is calculated by the following formula, which is based on running poikilotherms ( Buddenbrock, 1934 cited in Peters, 1983):

$c_{m}=\left( 0.17W^{0.75}+3.4 W \right)$ (eq. 13).

The cost of moving during the time *t_m_* (*t_m_* ∙*c_m_*) is subtracted from an individual’s energy reserve. Based on *t_m_* and $v_{opt}$ , the total distance an individual covers at day *t* (*d_max_*) is determined:

$d_{max}=v_{opt}\cdot t_{m}$(eq. 14)

Next, the perceptual range of an individual is determined by means of the following relationship:

$d_{per}=301W+0.097$ (eq. 15)

For simplicity, this relationship is linear and based on the assumption that the smallest individual (0.01g) has a perceptual range of 0.10 m and the largest individual (3g) a perceptual range of 1m. The effect of this relationship has been tested (see sensitivity analysis, supplementary material part 5). Moreover, the positive relationship between body size and perceptual range or reaction distance has been illustrated over a wide range of taxa, including arthropods (supplementary information of (Pawar, Dell and Van M. Savage, 2012)).

The searching area of an individual is circular and its radius (*rad*, in m) is calculated by taking into account the total distance the individual has covered during the day and the individual’s perceptual range (see supplementary material part 2 for an explanation of the formula calculating *rad*):

$rad=\sqrt{\frac{{2\cdot d}_{max}\cdot d_{per}+\pi\cdot{d_{per}}^{2}}{\pi}}$ (eq. 16).

In order to avoid side-effects of applying the variable rad for a continuous landscape within a cellular landscape, a value drawn from the following distribution, $\left[ -0.5\cdot SL,0.5\cdot SL \right],$is added to *rad*.

##### Habitat Choice

As habitat choice is informed, an individual moves to the cell with the highest amount of resources within its searching area.

#### Growth

The applied growth model is the one described by West et al. (2001) (West, Brown and Enquist, 2001) for deterministic growth

$W=W_{max}\times\left\{ 1-\left[ 1-\left( \frac{W_{0}}{W_{max}} \right)^{1/4} \right]e^{{-at_{d}}/{{4W_{max}}^{1/4}}} \right\}^{4}$(eq. 17).

Within this formula, $W$refers to an individual’s actual mass (in kg) at developmental age *t_d_* (in days)*, W_max_* to an individual’s genetically inherited adult mass (in kg), *W_0_* to an individual’s egg mass (in kg) and *a* to a species specific constant. Here, *a* was fixed at 0.2 which is a realistic value for species with deterministic growth (West, Brown and Enquist, 2001). When applying the formula, $W$, *W_max_* and *W_0_* are converted to gram. According to West et al. (2001) (West, Brown and Enquist, 2001) the proportion of available energy that is devoted to growth (*A*) is described by

$A=1-\left( {\frac{W}{W}}_{max} \right)^{0.25}$ (eq. 18).

In ideal conditions, an individual’s total amount of assimilated energy during one day (*E_a_*) should correspond to 0.55 ∙ *imax*. Here, we assume that an individual will only increase its developmental age by one day if *A* ∙ 0.55 ∙ *i_max_* Joules is still available within its energy reserve. Otherwise, its developmental age is not increased by one but by *E_r_*/( *A* ∙ 0.55 ∙ *i_max_*) with *E_r_* being the energetic content of an individual’s energy reserve. This value is larger than or equal to 0 and smaller than 1. This implies that individuals experiencing a shortage in energy will grow at a slower rate than individuals developing under ideal conditions.

#### Maturation

Juveniles reaching 99 percent of their adult mass (*W_max_*) mature.

#### Reproduction

During reproduction, the relationship between total clutch size (*CS*, in kg) and mass (*W*, in kg) is determined by the following equation which is based on aquatic poikilotherms (Blueweiss *et al.*, 1978)

$CS=0.158W^{0.92}$ (eq. 19).

However, an adult is only allowed to produce a clutch of this size if it has collected enough energy within its energy reserve and when it is located within a suitable patch. The entire clutch is deposited within that single patch, simultaneously. The required energy to produce such a clutch is calculated by

$E_{CS}=CS\cdot7\cdot{10}^{6}\frac{J}{kg}+N\cdot E_{i}$ (eq. 20).

Here, 1 kg of wet tissue is considered to have an energetic content of 7∙10^6^ Joules (Peters, 1983). For simplicity, the number of eggs per clutch (*N*) is assumed to be fixed at 15. Further, each newly laid egg is provisioned with the necessary energy to survive its first day (*E_i_*, in Joules). The mass of a newly laid egg (*W_0_*, in kg) is calculated as follows:

$W_{0}=\frac{CS}{N}$ (eq. 21).

Also, we guarantee that the adult size of an individual is never smaller than the size it obtained during the egg stage (i.e. $W_{0}\leq W$). Such contradictions might occur as the egg size of an individual depends on the adult size of its parent, not on its own adult size.

## Coupled versus decoupled model

To determine the importance of size-dependent movement, two different models were created: a coupled and a decoupled model. In the coupled model, speed of movement ($v_{opt}$) and perceptual range both increase with body size. The decoupled model represents a null model in which body size, speed of movement and perceptual range were unlinked. Body size and speed of movement were unlinked by resampling an individual’s speed of movement each day from the uniform range [0.0106, 0.0557]. Here, 0.0106 corresponds to the optimal speed of the smallest adult individual (0.01 g) and 0.0557 to the optimal speed of the largest adult individual (3 g). Also, the perceptual range of an individual is no longer increasing with body size, but instead sampled daily from the uniform distribution [0.1 m, 1 m]. 0.1 m corresponds to the perceptual range of the smallest adult individual (0.01 g) and 1m to the perceptual range of the largest adult individual (3 g). We chose to sample from a uniform distribution rather than from an evolved scenario in the decoupled model to avoid any skewness and bias in the randomization. As the cost of movement is based on the total movement time and not total distance, it is unaffected by the decoupling.

*Table S4.1: Overview and definition of all parameters. In case a parameter is fixed, this value is given. In case a parameter depends on an individual’s size, the corresponding allometric rule is given.*

| Parameter | Unit | Definition | Fixed value or equation |
| --- | --- | --- | --- |
| *NND* | Number of cells | Nearest neighbor distance | [0,10] |
| *t* | day | Time since the start of the simulation |  |
| *R_x,y_* | Joule | Total energetic content of resource tissue present within cell_x,y_ |  |
| *K* | Joule | Carrying capacity of resource per cell | 2000 Joules |
| *r* |  | Growth speed of the resource | 0.1, 0.5 or 0.9 |
| *E_nc_* | Joule | Fixed amount of non-consumable resource energy per cell | 1 Joule |
| *W_max_* | Kg | Adult size of an individual |  |
| *W* | Kg | Mass of an individual at developmental age *t_d_* | eq. 17 |
| *W_0_* | Kg | Egg mass of an individual | eq. 21 |
| *IR* | J/s | Ingestation rate of an individual with mass *W* | eq. 4 |
| *i_max_* | Joule | An individual’s maximum ingestible amount of energy at time *t* | eq. 5 |
| *t_f_* | seconds | Time devoted per day to consumption | 15 hours |
| *E_c_* | Joule | Energy being consumed by an individual at time *t* | eq. 6 |
| *E_a_* | Joule | Energy being assimilated by an individual at time *t* | eq. 7 |
| *M* | J/s | The standard metabolic rate of an individual with size *W* | eq. 8 |
| *M_day_* | J | Total standard metabolic rate costs for an individual at time *t* | eq. 9 |
| *t_m_* | seconds | Time devoted to movement at time *t* | eq. 12 |
| *p* | - | Probability of moving at time *t* | eq. 10 |
| *v_opt_* | m/s | Average speed of movement for individual with size *W* | eq. 11 |
| *d_max_* | meter | Maximum distance an individual can cover at time *t* | eq. 14 |
| *d_per_* | meter | Perceptual range of an individual | eq. 15 |
| *rad* | meter | Radius of searching area at time *t* | eq. 16 |
| *c_m_* | Joule | Cost of movement at time *t* | eq. 13 |
| *q* | 1/day | Immigration rate | 1 per 10 days |
| *a* | - | Species specific constant within formula of West et al. (2001)(West, Brown and Enquist, 2001) | 0.2 |
| *t_d_* | day | An individual’s developmental age |  |
| *A* | - | Proportion of available energy being devoted to growth at time t | eq. 18 |
| *E_r_* | Joule | Energy available within energy reservoir at time t |  |
| *CS* | Kg | Total clutch size of an individual with size W | eq. 19 |
| *E_CS_* | Joule | Required energy to produce a clutch of size CS | eq. 20 |
| *N* | - | Number of eggs within one clutch | 15 |
| *SL* | m | Side length of a cell within the landscape | 0.25 |

*Table S4.2: An overview of the total runtime per simulation within the decoupled model.*

|  | NND 0 | NND 1 | NND 2 | NND 3 | NND 4 | NND 5 | NND 6 | NND 7 | NND 8 | NND 9 | NND 10 |
| --- | --- | --- | --- | --- | --- | --- | --- | --- | --- | --- | --- |
| Growth speed 0.1 | 2000 | 2000 | 2000 | 2000 | 2000 | 2000 | 2000 | 2000 | 2000 | 2000 | 2000 |
| Growth speed 0.5 | 2000 | 2000 | 1500 | 1500 | 1500 | 2000 | 2000 | 2000 | 2000 | 2000 | 2000 |
| Growth speed 0.9 | 2000 | 1000 | 1000 | 1000 | 1000 | 1000 | 1500 | 2000 | 2000 | 2000 | 2000 |

## References

Berg, M. P., Kiers, E. T., Driessen, G., van der HEIJDEN, M., Kooi, B. W., Kuenen, F., Liefting, M., Verhoef, H. a. and Ellers, J. (2010) ‘Adapt or disperse: understanding species persistence in a changing world’, *Glob Chang Biol*, 16(2), pp. 587–598. doi: 10.1111/j.1365-2486.2009.02014.x.

Blueweiss, L., Fox, H., Kudzma, V., Nakashima, D., Peters, R. and Sams, S. (1978) ‘Relationships between body size and some life history parameters’, *Oecologia*, 37, pp. 257–272.

Brown, J. H., Gillooly, J. F., Allen, A. P., Savage, V. M. and West, G. B. (2004) ‘Toward a metabolic theory of ecology’, *Ecology*, 85(7), pp. 1771–1789. doi: 10.1890/03-9000.

Buddenbrock, W. V. (1934) ‘Uber die kinetische und statische Leistung grosser und kleiner Tiere und ihre bedeutung für dem Gesamtstoffwechsel’, *Naturwissenschaft*, 22, pp. 675–680.

Davies, K. F., Margules, C. R. and Lawrence, J. F. (2000) ‘Which traits of species predict population declines in experimental forest fragements?’, *Ecology*, 81(5), pp. 1450–1461. doi: 10.1890/0012-9658(2000)081[1450:WTOSPP]2.0.CO;2.

Delgado, M. M., Barton, K. A., Bonte, D. and Travis, J. M. J. (2014) ‘Prospecting and dispersal: their eco-evolutionary dynamics and implications for population patterns’, *Proceedings of the Royal Society B: Biological Sciences*, 281(1778), pp. 20132851–20132851. doi: 10.1098/rspb.2013.2851.

Fahrig, L. (2003) ‘Effects of Habitat Fragmentation on Biodiversity’, *Annual Review of Ecology, Evolution, and Systematics*, 34(2003), pp. 487–515. doi: 10.1146/132419.

Gagné, S. A., Proulx, R. and Fahrig, L. (2008) ‘Testing Holling’s textural-discontinuity hypothesis’, *Journal of Biogeography*, 35(12), pp. 2149–2150. doi: 10.1111/j.1365-2699.2008.02031.x.

Grimm, V., Berger, U., Bastiansen, F., Eliassen, S., Ginot, V., Giske, J., Goss-Custard, J., Grand, T., Heinz, S. K., Huse, G., Huth, A., Jepsen, J. U., Jørgensen, C., Mooij, W. M., Müller, B., Pe’er, G., Piou, C., Railsback, S. F., Robbins, A. M., Robbins, M. M., Rossmanith, E., Rüger, N., Strand, E., Souissi, S., Stillman, R. A., Vabø, R., Visser, U. and DeAngelis, D. L. (2006) ‘A standard protocol for describing individual-based and agent-based models’, *Ecological Modelling*, 198(1–2), pp. 115–126. doi: 10.1016/j.ecolmodel.2006.04.023.

Grimm, V., Berger, U., DeAngelis, D. L., Polhill, J. G., Giske, J. and Railsback, S. F. (2010) ‘The ODD protocol: A review and first update’, *Ecological Modelling*. Elsevier B.V., 221(23), pp. 2760–2768. doi: 10.1016/j.ecolmodel.2010.08.019.

Hemmingsen, A. M. (1960) ‘Energy metabolism as related to body size and respiratory surfaces, and its evolution’, *Reports of the Steno Memorial Hospital and Nordinsk Insulin Laboratorium*, 9, pp. 6–110.

Henry, R. C., Coulon, A. and Travis, J. M. J. (2015) ‘Dispersal asymmetries and deleterious mutations influence metapopulation persistence and range dynamics’, *Evolutionary Ecology*. Springer International Publishing, 29(6), pp. 833–850. doi: 10.1007/s10682-015-9777-4.

Holling, C. S. (1992) ‘Cross-Scale Morphology, Geometry, and Dynamics of Ecosystems’, *Ecological Monographs*, 62(4), pp. 447–502. doi: 10.2307/2937313.

Moses, M. E., Hou, C., Woodruff, W. H., West, G. B., Nekola, J. C., Zuo, W. and Brown, J. H. (2008) ‘Revisiting a model of ontogenetic growth: estimating model parameters from theory and data.’, *The American naturalist*, 171(5), pp. 632–645. doi: 10.1086/587073.

Oksanen, A. J., Blanchet, F. G., Kindt, R., Legendre, P., Minchin, P. R., Hara, R. B. O., Simpson, G. L., Solymos, P., Stevens, M. H. H. and Wagner, H. (2018) ‘vegan: Community Ecology Package’. doi: ISBN 0-387-95457-0.

Pawar, S., Dell, A. I. and Van M. Savage (2012) ‘Dimensionality of consumer search space drives trophic interaction strengths’, *Nature*. Nature Publishing Group, 486(7404), pp. 485–489. doi: 10.1038/nature11131.

Peters, R. H. (1983) *The ecological implications of body size*, *Cambridge studies in ecology*. Edited by E. Beck, H. J. B. Birks, and E. F. Connor. Cambridge: Cambridge university press. Available at: http://books.google.com/books?id=OYVxiZgTXWsC.

Poethke, H. J. and Hovestadt, T. (2002) ‘Evolution of density- and patch-size-dependent dispersal rates.’, *Proceedings. Biological sciences / The Royal Society*, 269(1491), pp. 637–45. doi: 10.1098/rspb.2001.1936.

Raffaelli, D., Hardiman, A., Smart, J., Yamanaka, T. and White, P. C. L. (2016) ‘The textural discontinuity hypothesis: An exploration at a regional level. Shortened version: Exploring Holling’s TDH’, *Oikos*, 125(6), pp. 797–803. doi: 10.1111/oik.02699.

Ricklefs, R. E. (1974) ‘Energetics of reproduction in birds’, in Paynter, R. A. (ed.) *Avian energetics*. Cambridge, Massachusetts: Nuttall Ornithological Club, pp. 152–297.

Travis, J. M. J., Mustin, K., Benton, T. G. and Dytham, C. (2009) ‘Accelerating invasion rates result from the evolution of density-dependent dispersal’, *Journal of Theoretical Biology*, 259(1), pp. 151–158. doi: 10.1016/j.jtbi.2009.03.008.

Tscharntke, T. and Brandl, R. (2004) ‘Plant- Insect Interactions in Fragmented Landscapes’, *Annual Review of Entomology*, 49(1), pp. 405–430. doi: 10.1146/annurev.ento.49.061802.123339.

Wang, S. and Loreau, M. (2014) ‘Ecosystem stability in space: α, β and γ variability’, *Ecology Letters*. Edited by A. Liebhold, 17(8), pp. 891–901. doi: 10.1111/ele.12292.

West, G. B., Brown, J. H. and Enquist, B. J. (2001) ‘A general model for ontogenetic growth’, *Nature*, 413(6856), pp. 628–631. doi: 10.1038/35098076.
