# Supplementary material for: Size‐dependent movement explains why bigger is better in fragmented landscapes
Source: Ecol Evol. 2018 Oct 23;8(22):10754–67. doi: 10.1002/ece3.4524 (PMC6262741; doi:10.1002/ece3.4524)
Supplement: Supplementary file 5 [file ECE3-8-10754-s005.docx]

# Supplementary material part 5

# Sensitivity analysis

Table 1: This table represents an overview of the sensitivity analyses.

| *Parameter* | *Default setting* | *Extra tested values or functions* |
| --- | --- | --- |
| *K* | 2000 | 1000, 3000 |
| *N* | 15 | 2, 50 |
| *t_f_* | 15 hours | 10 hours, 20 hours |
| *t_m_* | 1h | 30 min, 2hours |
| *i* | 0.1 | 0 |
| *d_per_* | 301 *W* + 0.097 | 133.779*W* + 0.0987, 331.104*W* +0.00669 |

The original model: Coupled


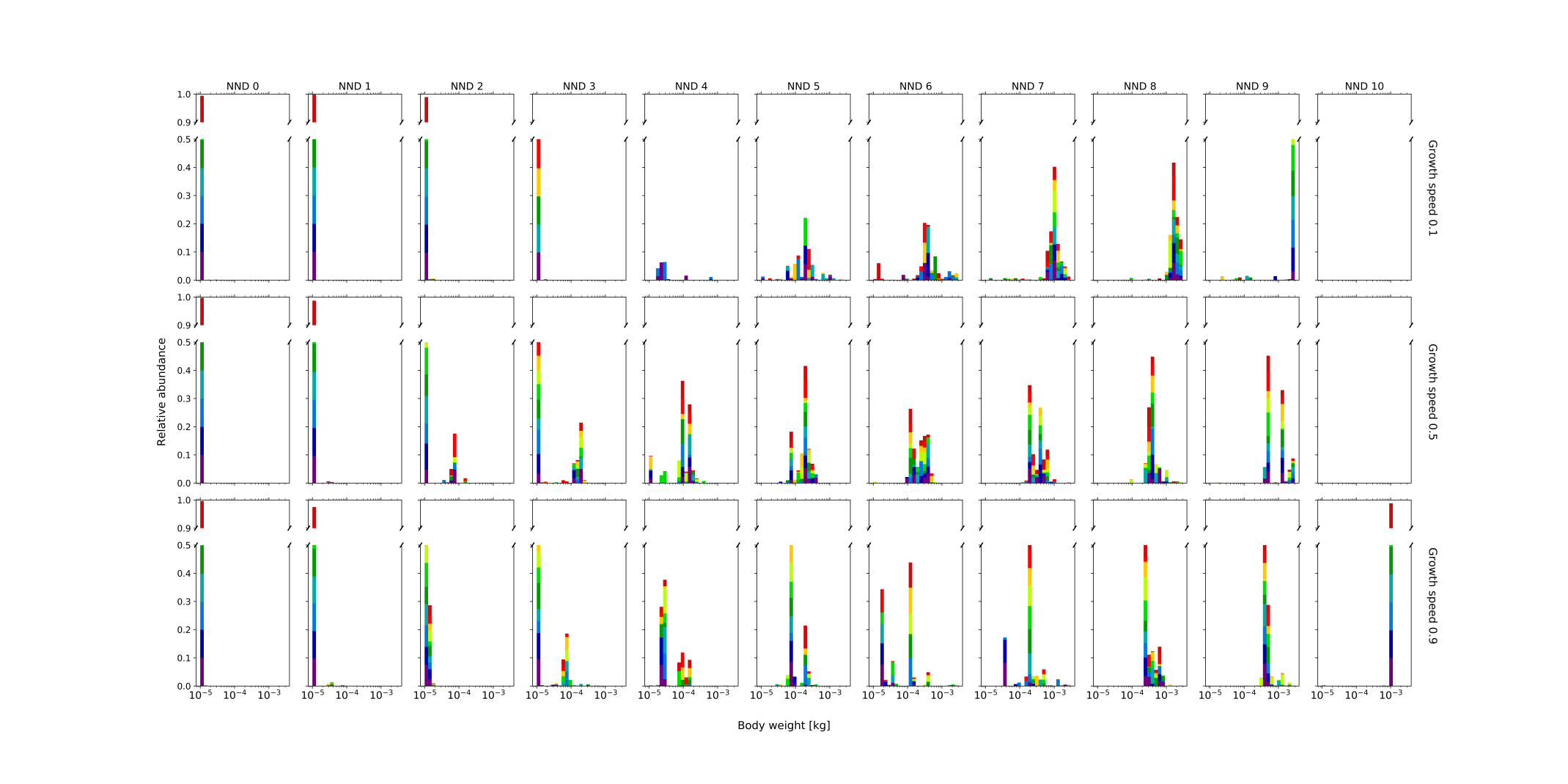


The original model: decoupled


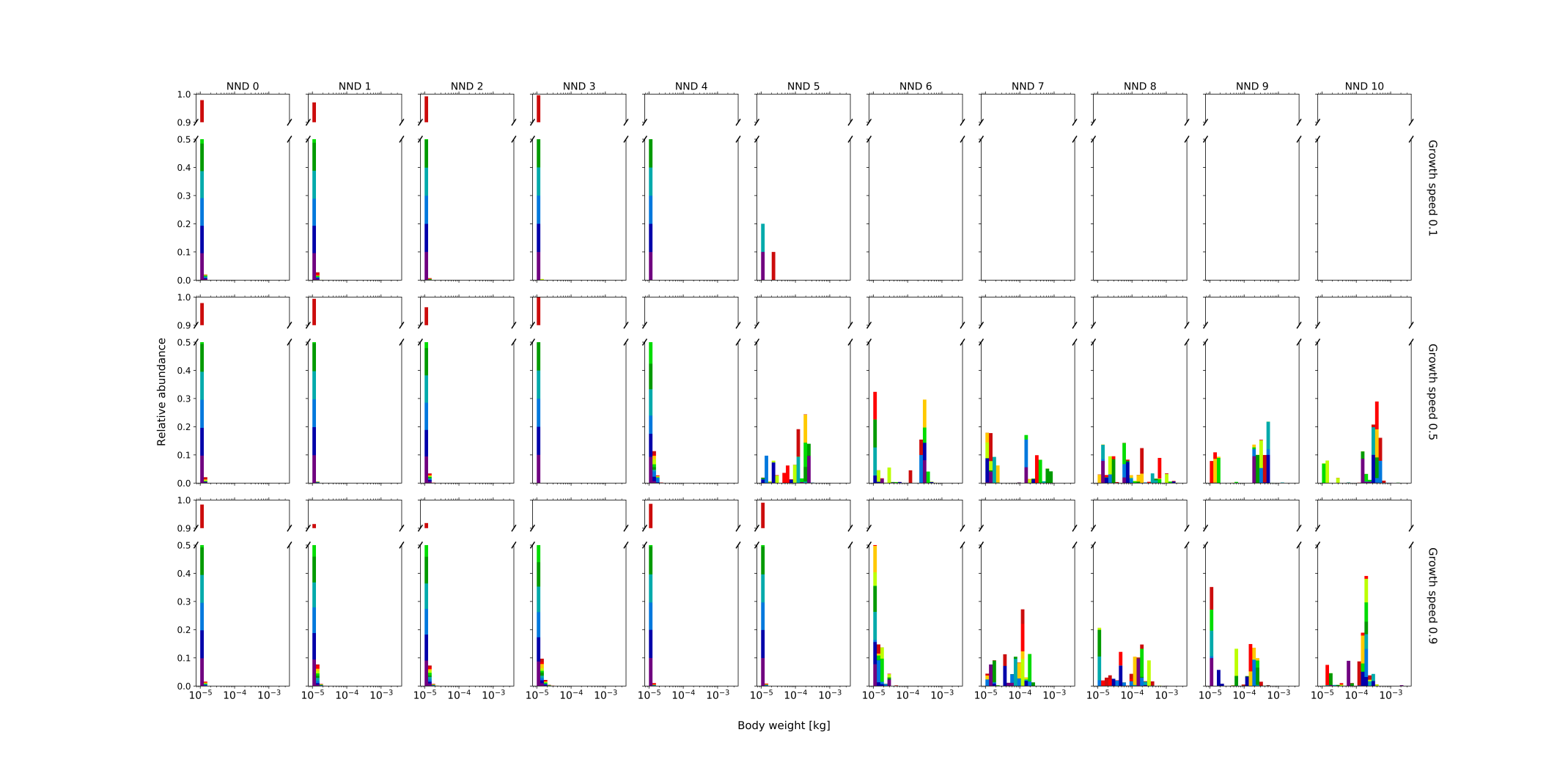


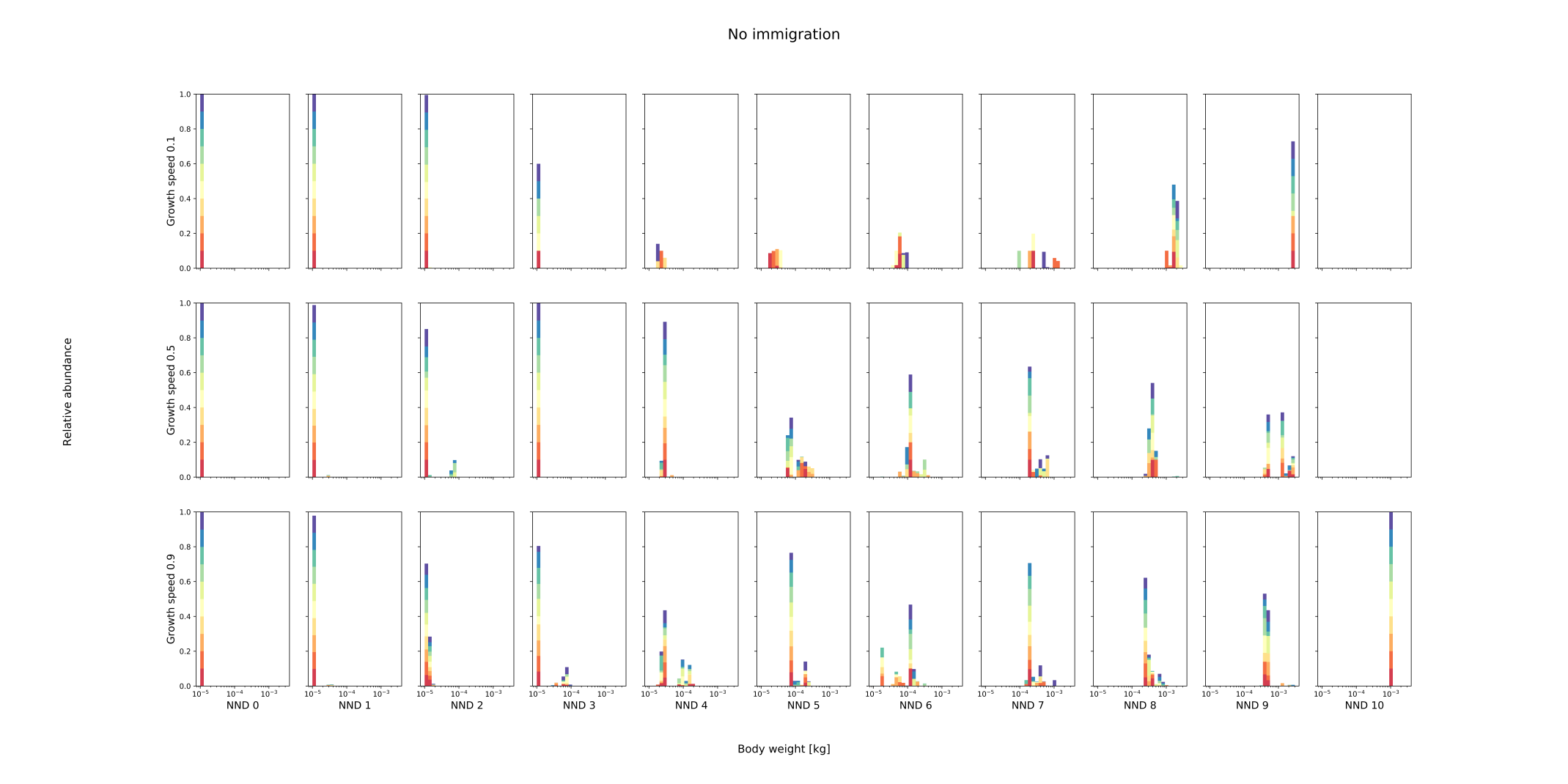


No immigration within original coupled model: Simulations with 2 optima continue to exist except for NND 3 and growth speed 0.5.


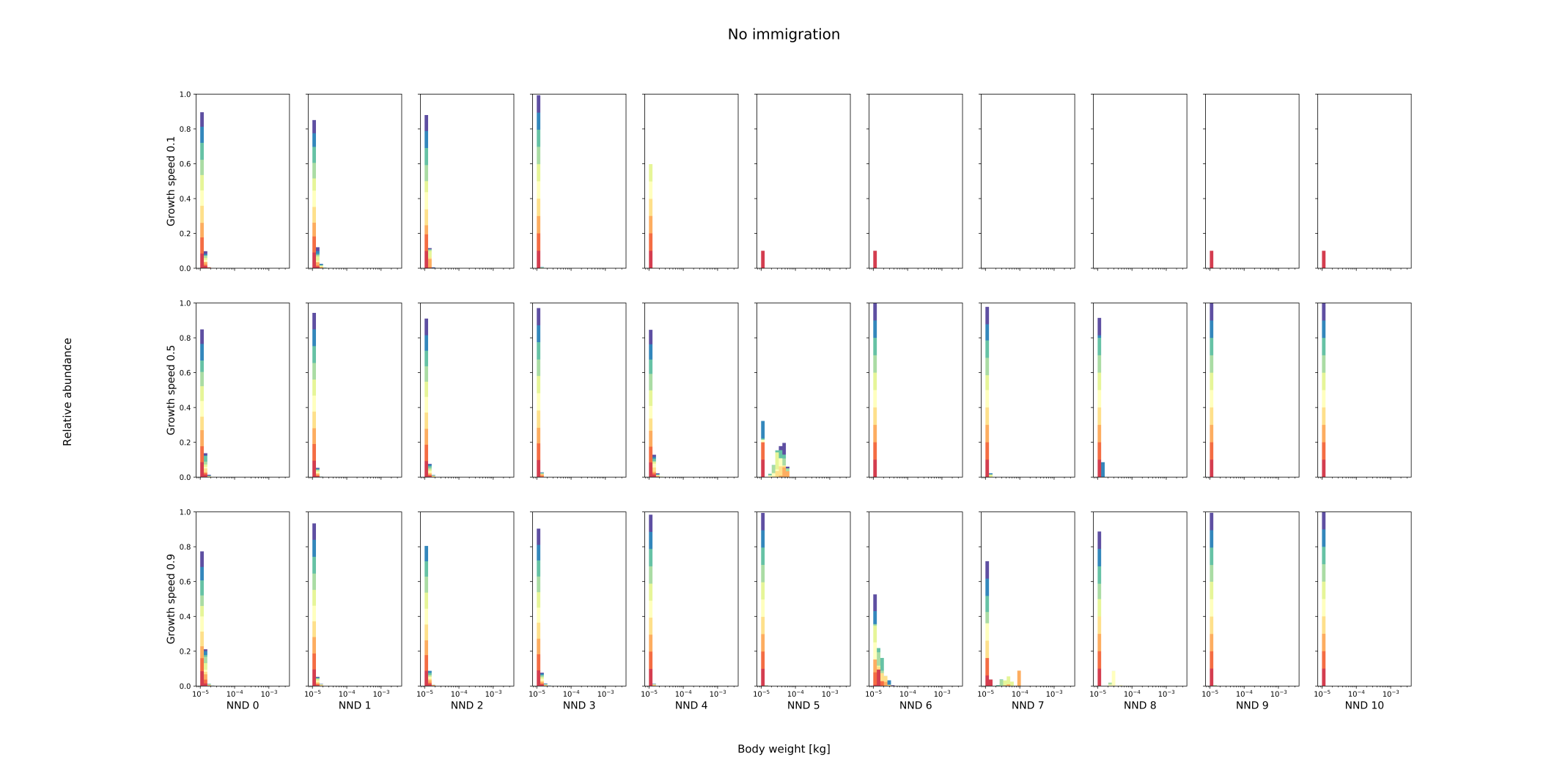


No immigration within original decoupled model: Always selection in favor of smallest individuals. This proves that immigration results in higher genetic load, preventing the population from reaching its optimum body size distribution. (All simulations ran for 1000 time steps.)


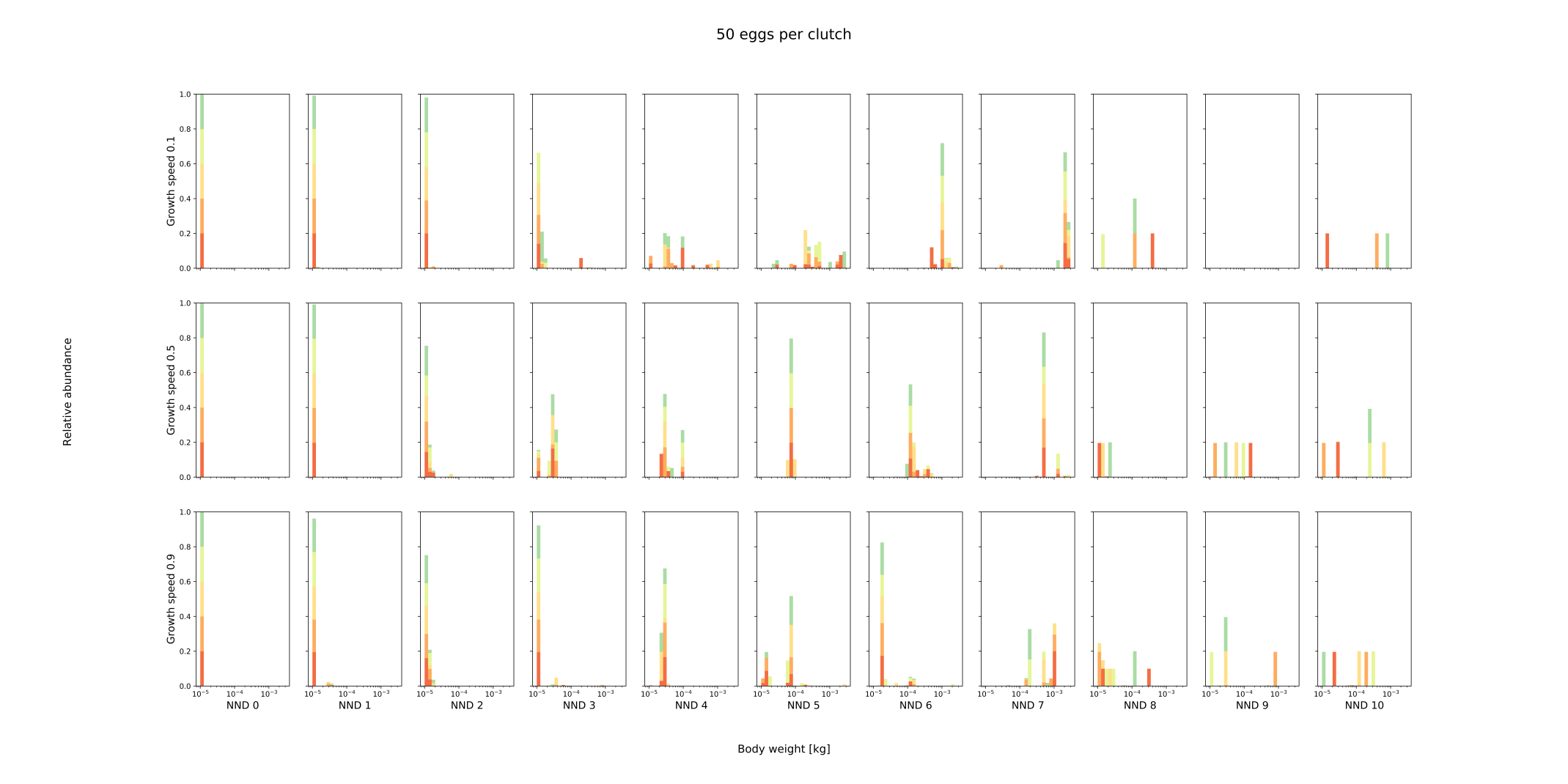


Fifty eggs per clutch within the coupled model: From NND 8 onwards, simulations are not stable and consist out of immigrants (sinks). The higher number of eggs results in stronger competition for resources and therefore, extinction.
The overall pattern within the original model (from NND 0 to 10) corresponds with the pattern observed here (from NND 0 to 7).


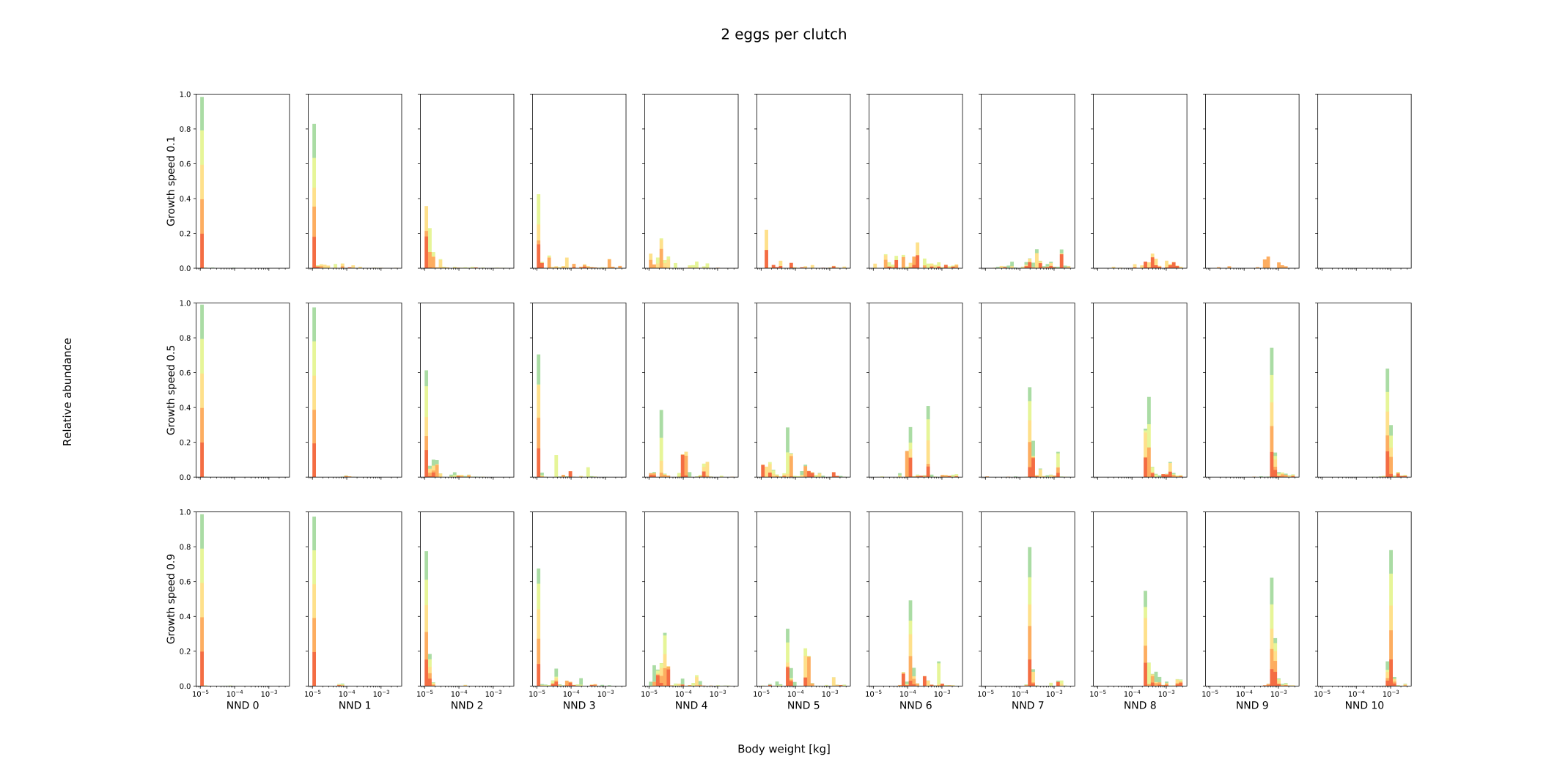


Two eggs per clutch within the coupled model: Simulations with NND 10 and growth speed 0.5 do now survive whereas they didn’t within the original model. Fewer eggs per clutch results in lowered competition for resources and therefore, higher survival chances. When growth speed of the resource is minimal (0.1) and NND intermediate, small and large individuals appear to coexist within the landscape. As large individuals increase much slower in number, they are not able to dominate the population. Therefore, both strategies fluctuate in abundance according to the availability of resources within the landscape (see below for example). When NND 9 and growth speed 0.1, the largest individuals have too few offspring per clutch to persist within the landscape.


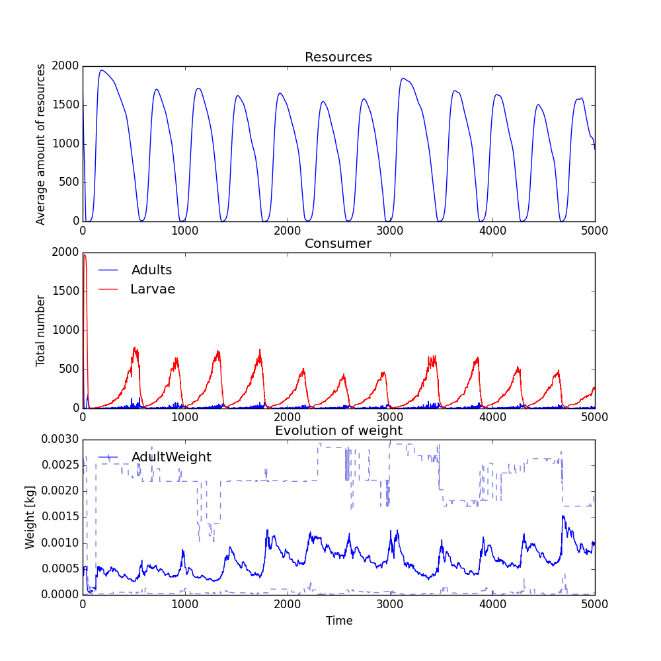

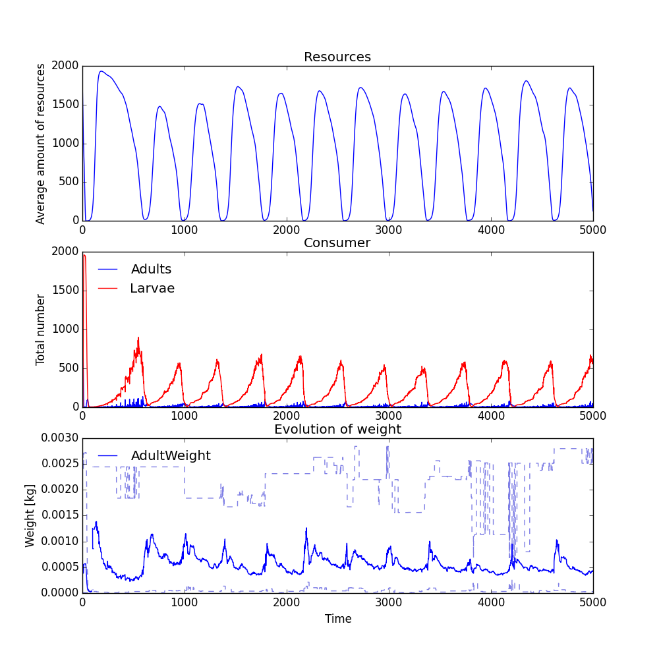

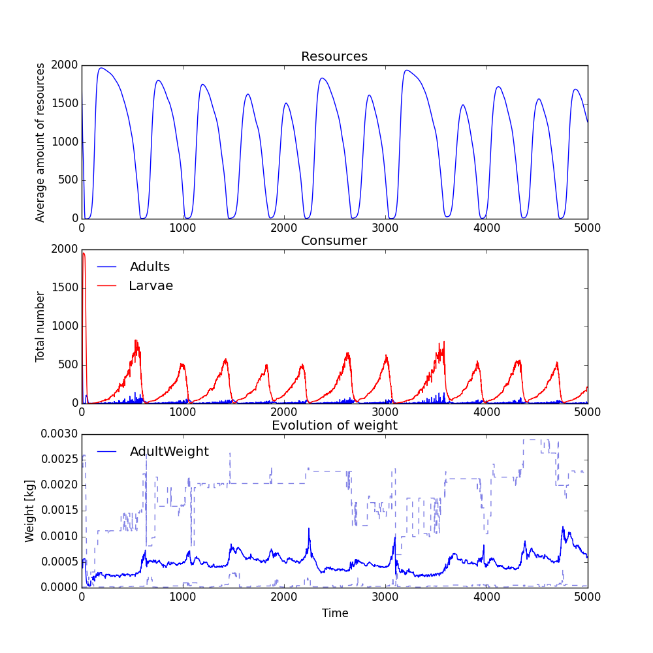


Two eggs per clutch within the coupled model: The outcome of three simulations with NND7 and growth speed 0.1.


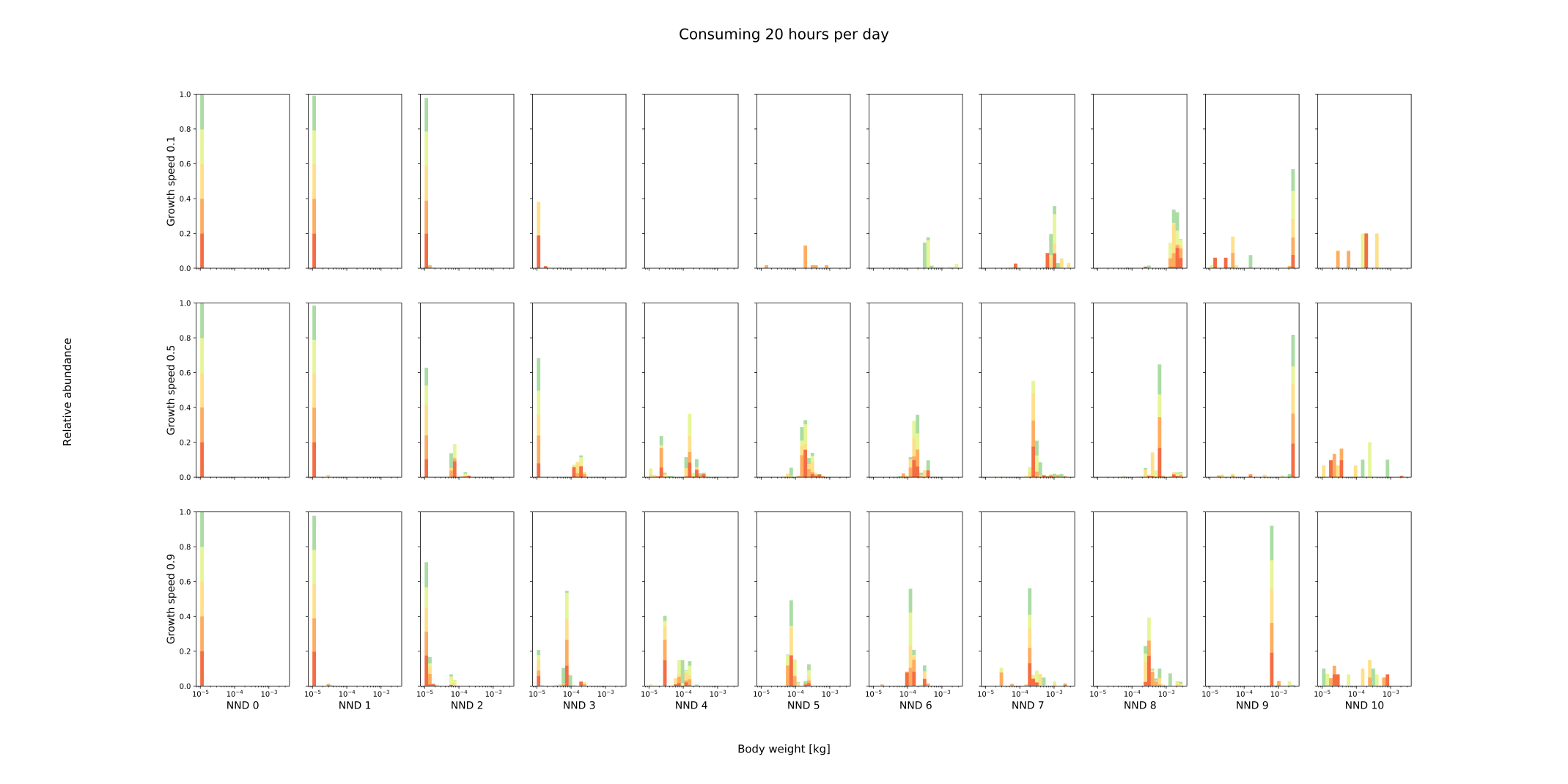


Consuming 20 hours per day within the coupled model: As individuals do eat more per day, competition for resources is stronger than within the original model. As such, when growth speed is lowest and NND intermediate (3 to 6) more simulations go extinct. Also, when NND 10, populations cannot persist without immigration from elsewhere.


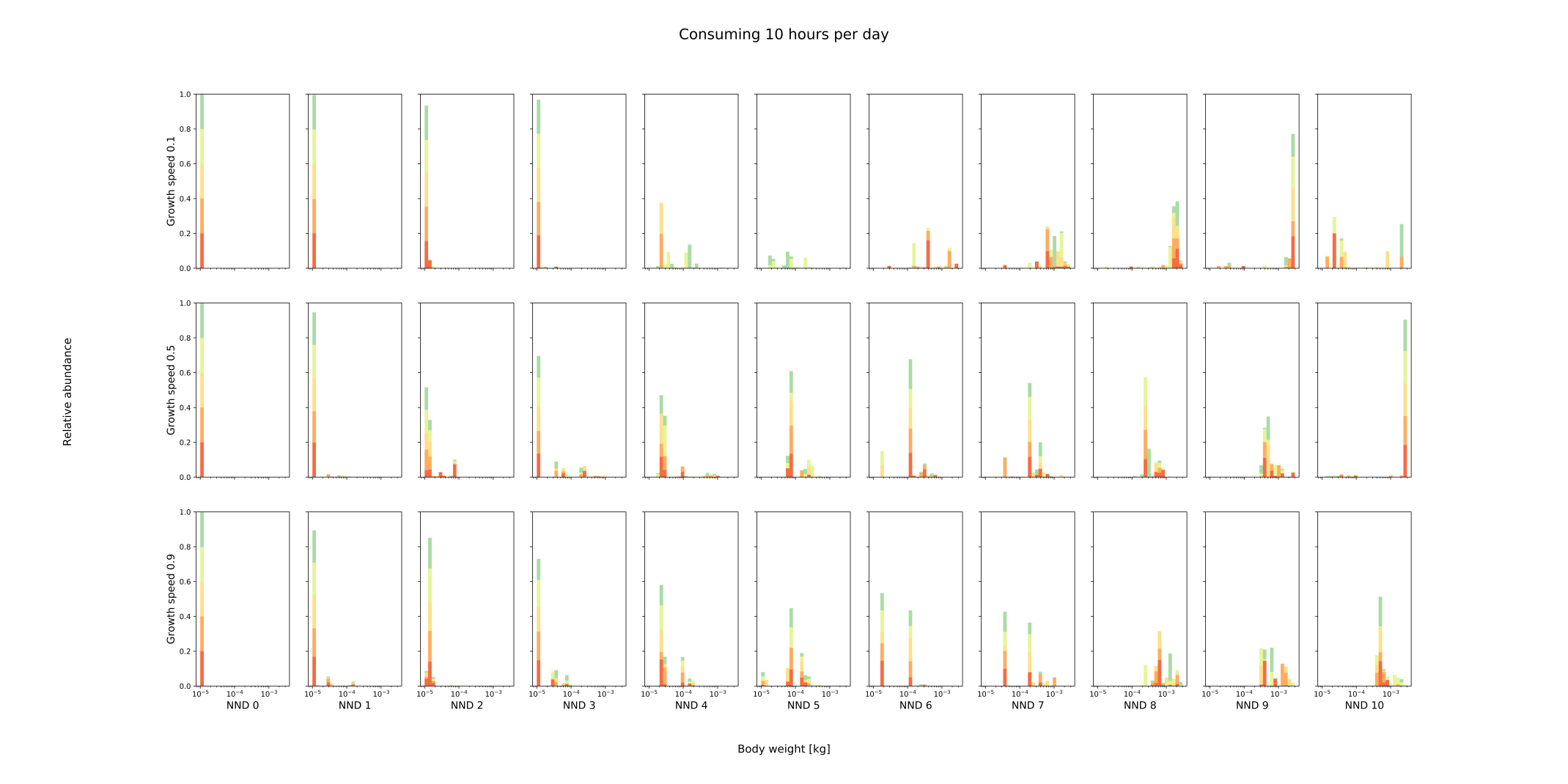


Consuming 10 hours per day within the coupled model:

Competition is lowered as less food is eaten per day. Therefore, when NND 10 and growth speed of the resource 0.5, stable populations can persist.


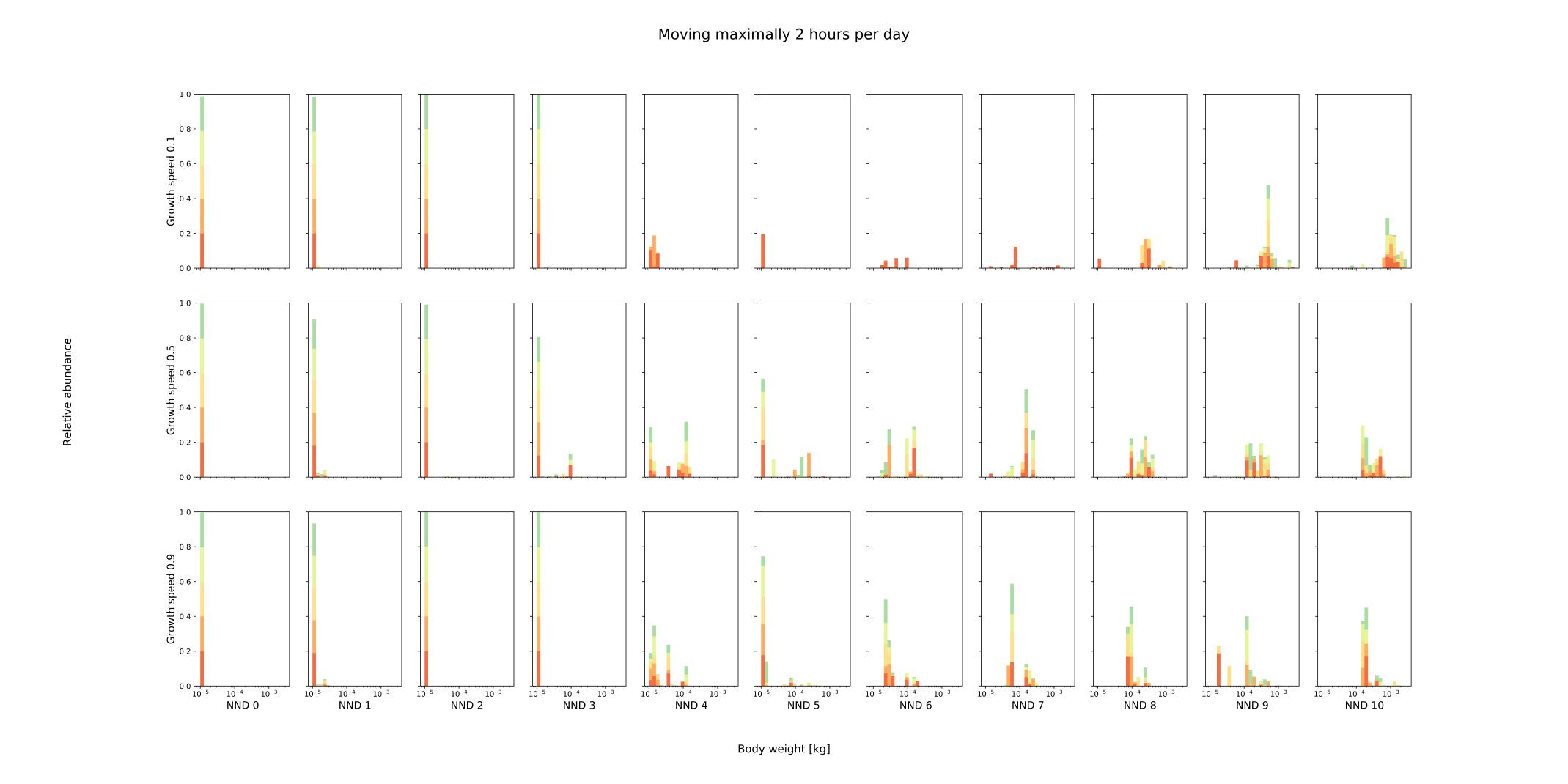


Moving maximally two hours per day within the coupled model: As smaller individuals can move further, individuals are smaller than within the original model. However, the general trend of individuals becoming larger with increasing NND and decreasing growth speed, is still observed. (All simulations ran for 4000 time steps.)


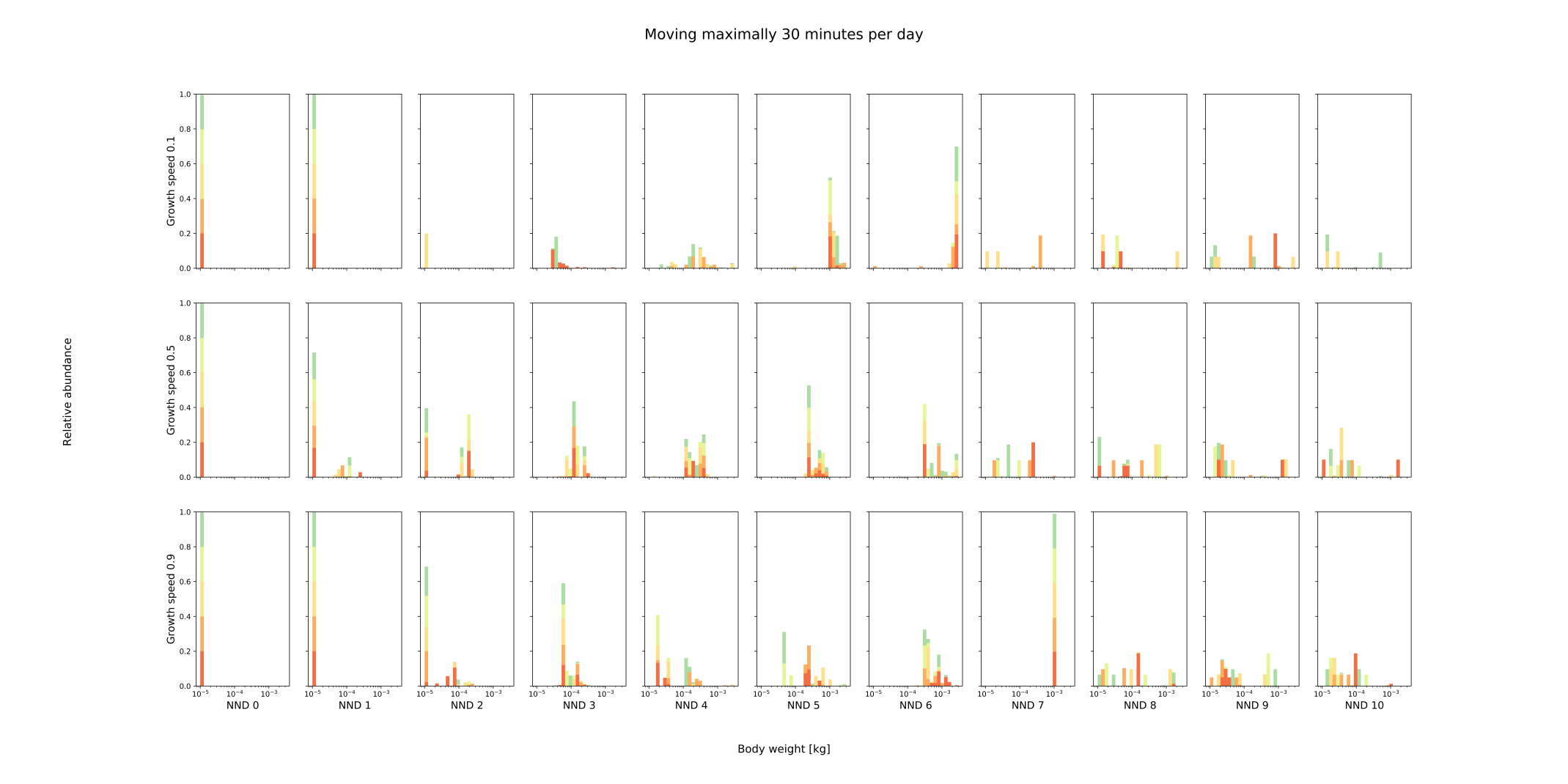


Moving maximally 30 minutes per day within the coupled model: As all individuals have a smaller foraging area, no stable populations can persist within a landscape with NND 8 or higher (sinks). The general trend observed within the original model from NND0 to NND 10 is now observed from NND 0 to NND 7.


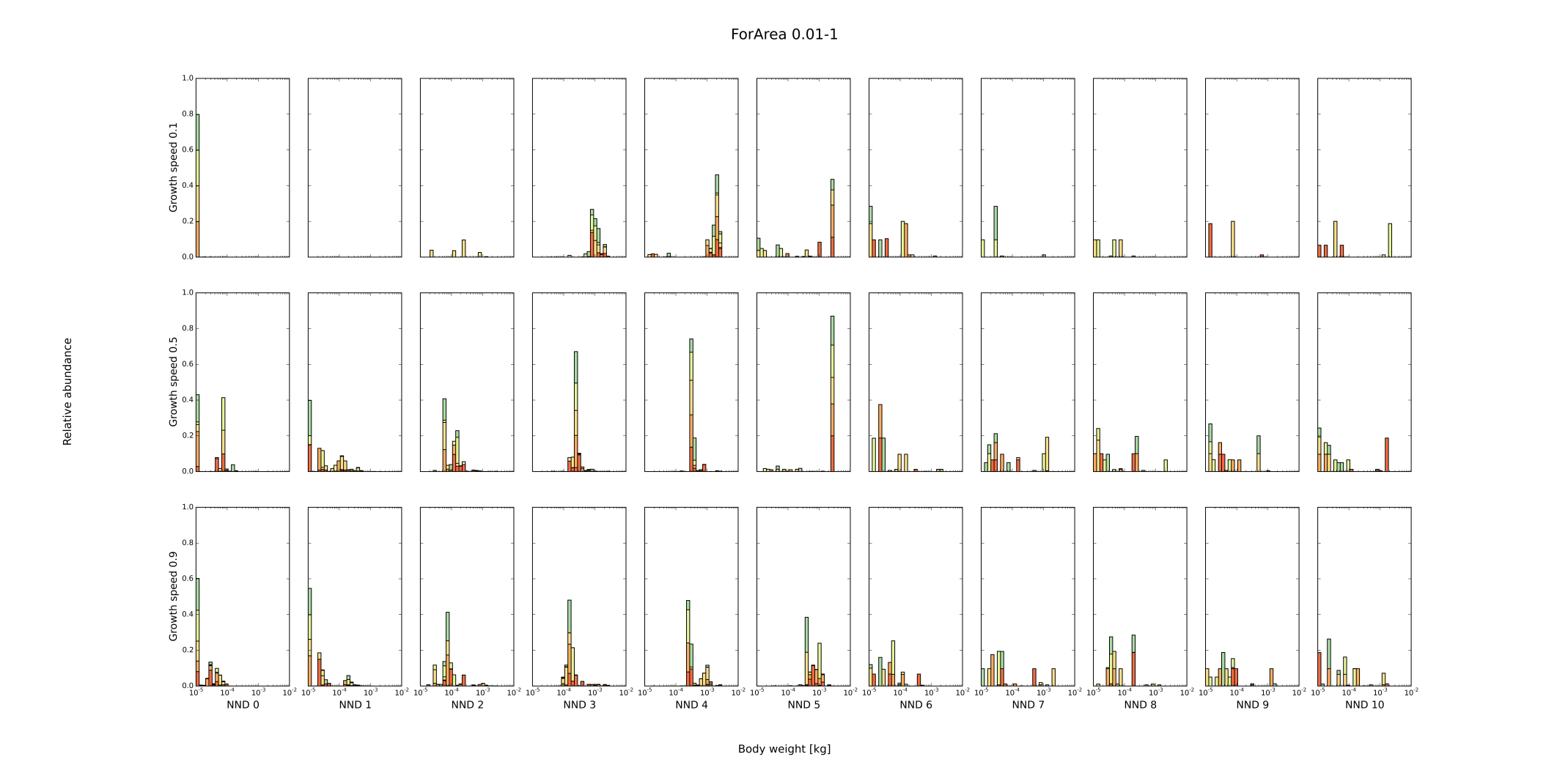


Smallest individuals (± 1 cm length) have a perceptual range of 1 cm (instead of 10 cm) within the coupled model: From NND 6 onwards, populations are not stable (persistence of population based on immigrants). The pattern of NND 0 to NND 5 resembles the pattern of NND 3 to NND 9 within the original model. As the smallest individuals move less, they are no longer able to dominate the population from NND0 to NND2 as they do within the original model. This even results in increased extinction chance when growth speed 0.1.


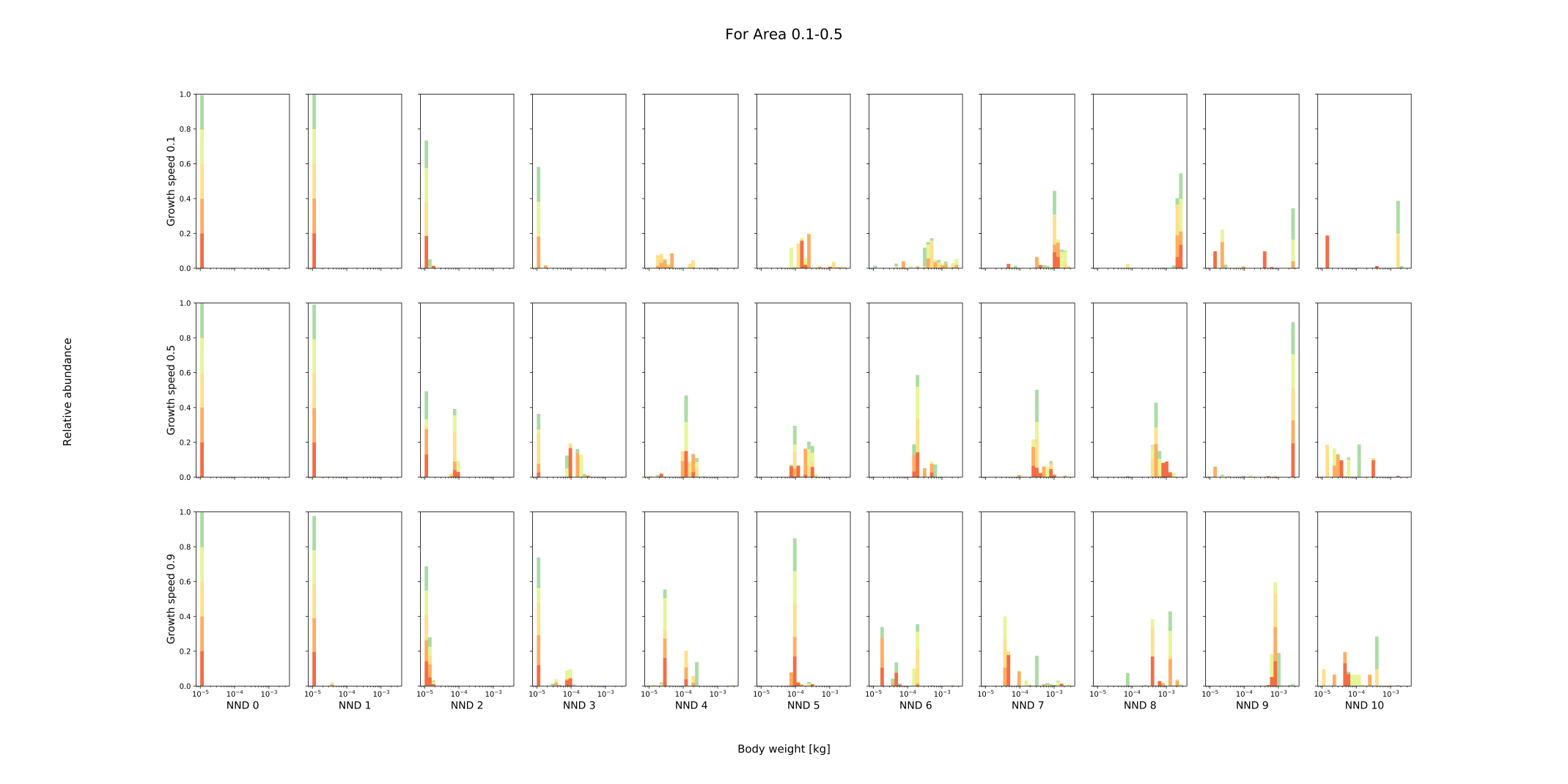


Largest individuals (± 10 cm length) have a perceptual range of 0.5 m (instead of 1 m) within the coupled model: As large individuals have a smaller perceptual range, they can no longer persist within a landscape with NND 10 and NND 9 with growth speed 0.1. The populations living in these landscapes are not stable (sinks).


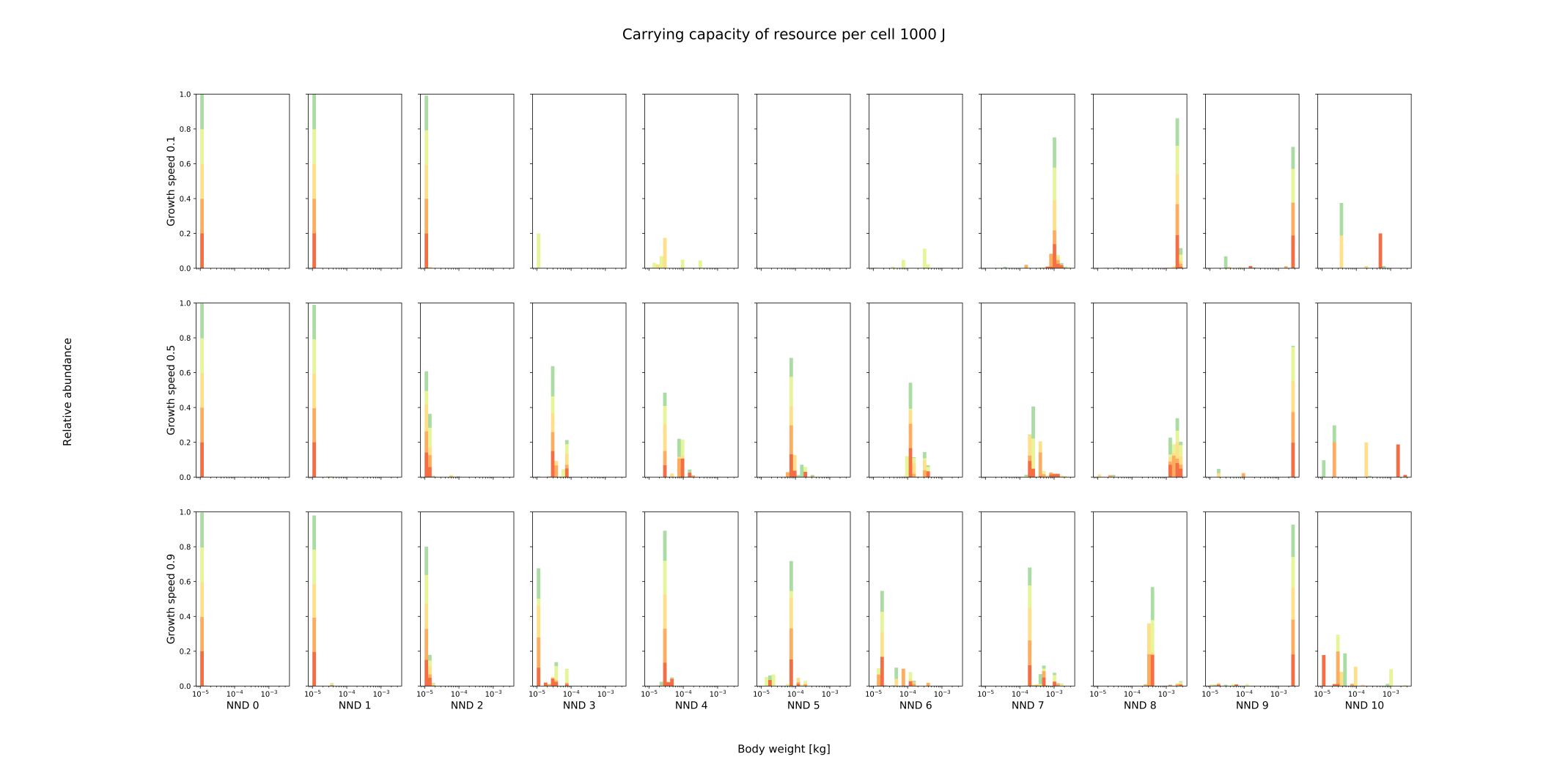


Carrying capacity of resource per cell 1000J within the coupled model: No stable equilibrium of large individuals for scenario with NND 10 and r 0.9 due to too few available resources. Also, extinction is higher for intermediate isolation when growth speed is low.


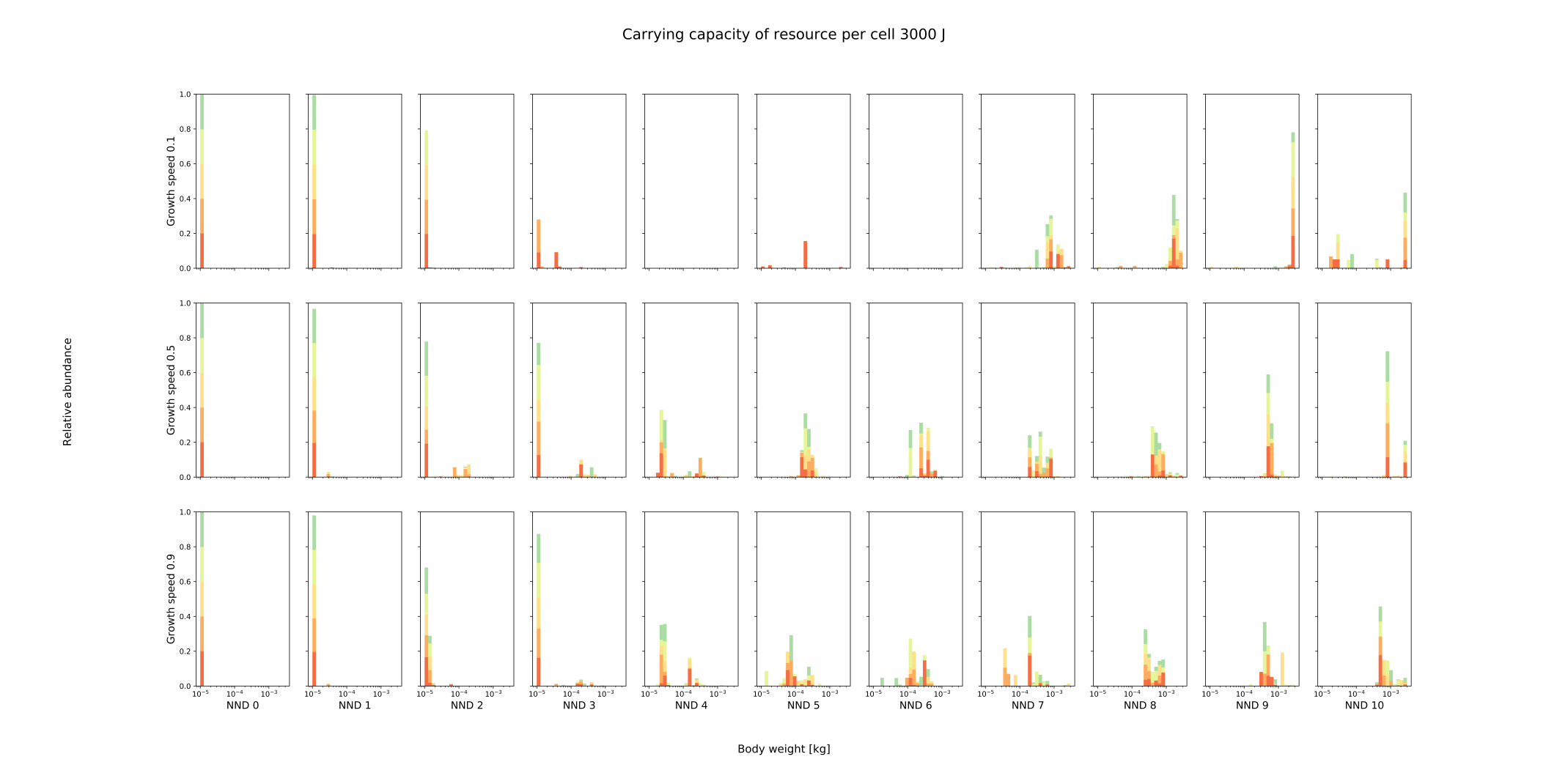


Carrying capacity of resource per cell 3000J within the coupled model: Populations within landscapes with growth speed 0.5 and NND 10 do also survive. (All simulations ran 4000 time steps.)
